# Digital Control of a Superconducting Qubit Using a Josephson Pulse Generator at 3 K

**DOI:** 10.1103/prxquantum.3.010350

**Published:** 2020-03

**Authors:** L. Howe, M. A. Castellanos-Beltran, A. J. Sirois, D. Olaya, J. Biesecker, P. D. Dresselhaus, S. P. Benz, P. F. Hopkins

**Affiliations:** 1 National Institute of Standards and Technology, Boulder, Colorado 80305, USA; 2 University of Colorado, Boulder, Colorado 80309, USA

## Abstract

Scaling of quantum computers to fault-tolerant levels relies critically on the integration of energy-efficient, stable, and reproducible qubit control and readout electronics. In comparison to traditional semiconductor-control electronics (TSCE) located at room temperature, the signals generated by rf sources based on Josephson-junctions (JJs) benefit from small device sizes, low power dissipation, intrinsic calibration, superior reproducibility, and insensitivity to ambient fluctuations. Previous experiments to colocate qubits and JJ-based control electronics have resulted in quasiparticle poisoning of the qubit, degrading the coherence and lifetime of the qubit. In this paper, we digitally control a 0.01-K transmon qubit with pulses from a Josephson pulse generator (JPG) located at the 3-K stage of a dilution refrigerator. We directly compare the qubit lifetime *T*_1_, the coherence time $T_{2}^{*}$, and the thermal occupation *P*_th_ when the qubit is controlled by the JPG circuit versus the TSCE setup. We find agreement to within the daily fluctuations of ±0.5 *μ*s and ±2 *μ*s for *T*_1_ and $T_{2}^{*}$, respectively, and agreement to within the 1% error for *P*_th_. Additionally, we perform randomized benchmarking to measure an average JPG gate error of 2.1 × 10^−2^. In combination with a small device size (*<* 25 mm^2^) and low on-chip power dissipation (≪100 *μ*W), these results are an important step toward demonstrating the viability of using JJ-based control electronics located at temperature stages higher than the mixing-chamber stage in highly scaled superconducting quantum information systems.

## I. INTRODUCTION

Error-corrected quantum computers are projected to require large numbers, $𝓞\left(10^{6}\right)$, of qubits [1–4], placing stringent requirements on the per-qubit hardware overhead. Superconducting quantum circuits are a leading technology for scaling existing systems into the noisy intermediate-scale quantum (NISQ) era of ≳ 1000 qubits. In present systems, qubit gates and entangling operations are performed using shaped microwave pulses synthesized using instrumentation at room temperature [5]—here referred to as traditional semiconductor-control electronics (TSCE). Signals are routed into a dilution refrigerator (DR) to the approximately 0.01-K qubits and typically attenuated by 40–60 dB to suppress thermal noise on drive lines [6].

Limitations in cryogenic cooling power, TSCE power instability [7], and system complexity mandate a shift to miniaturize and enhance the stability and/or precision of waveform generation in superconducting quantum information systems. Recently, the pulse-shaping DACs and/or mixers used to generate qubit control signals have been successfully integrated at 3 K [8,9] and 100 mK [10] using cryogenic CMOS (cryoCMOS) technology. While this is an impressive step toward miniaturization and large-scale integration, a significant gap exists between these devices and scalable qubit control. Specifically, gate fidelity; power dissipation; and the accuracy, stability, and repeatability of the signals need improvement [7,11,12].

The scalability constraints of physical size and power consumption per channel may be satisfied by superconducting Josephson-junction (JJ) signal-generator circuits or the aforementioned cryoCMOS controllers. Comparable to cryoCMOS devices, JJ circuits have small device sizes (*<* 1 × 1 cm^2^) and very low on-chip power dissipation (≪100 *μ*W); while also leveraging the intrinsically calibrated nature of single-flux quantum (SFQ) pulses. This feature provides avenues for improving waveform quality and repeatability beyond what is achievable using semiconductor-based generators. Capitalizing on pulse-area quantization enables the use of JJ arrays to construct exceptionally stable and repeatable voltage sources from dc to a few gigahertz [13–16]. Similar devices are used to realize intrinsically accurate voltages for the international system of units and are disseminated worldwide as primary dc and ac voltage standards [17,18]. Furthermore, the use of SFQ pulses has been proposed as a scalable paradigm for digitally controlling qubits [19–21] and has recently been demonstrated with a SFQ driver and qubit circuit cofabricated on the same chip [22].

The primary limitation of SFQ operation proximal to quantum arrays is degradation of qubit lifetimes from quasiparticles created during pulse generation [23,24]. A solution that mitigates these quasiparticles must be implemented—such as physically separating the SFQ elements and qubits. In this work, we locate the JJ control circuitry on the 3-K DR stage to interrupt quasiparticle-qubit propagation. Similar to Ref. [22], we deliver sparse trains of pulses subresonantly to enact control—giving our device its name of the Josephson pulse generator (JPG). A 0.01-K bump-bonded multichip configuration [25,26] and/or the introduction of normal-metal quasiparticle traps can also be effective for quasiparticle mitigation [27,28].

The location of the cryogenic control electronics at a higher-temperature stage liberates physical volume at 0.01 K—commonly monopolized by the quantum array and readout hardware—and leverages higher cooling powers. This approach may also benefit from integration with cryoCMOS circuits by exploiting the advantages of cryoCMOS-implemented logic and/or memory elements [8–10]. The location of the control electronics at 3 K does increase the wiring complexity and the parasitic heat loads to the *<* 3 K stages; however, solutions are under development that demonstrate low thermal loading and crosstalk [29–31].

While the aforementioned merits of JJ-based sources [17] are expected to apply for qubit control, this work is the first validation of using JJ-based pulse generation at 3 K to control a 0.01-K qubit. Here, we show that the JPG does not adversely affect the qubit by separately measuring the qubit energy-relaxation time *T*_1_, the coherence time $T_{2}^{*}$, and the thermal occupancy *P*_th_ with both a TSCE setup and the JPG. Our findings show good agreement in all three metrics with each control setup. Additionally, we measure the JPG gate fidelity to be within an order of magnitude of the qubit coherence limit and provide discussion of future devices expected to yield coherence-limited gates.

## II. JPG-BASED QUBIT CONTROL

An input current evolving the JJ superconducting phase difference by 2*π* generates a voltage pulse the time-integrated area of which equals the magnetic flux quantum $\Phi_{0}\equiv h/2e$:

(1) $$\int Vdt=\Phi_{0}.$$

**FIG. 1.**
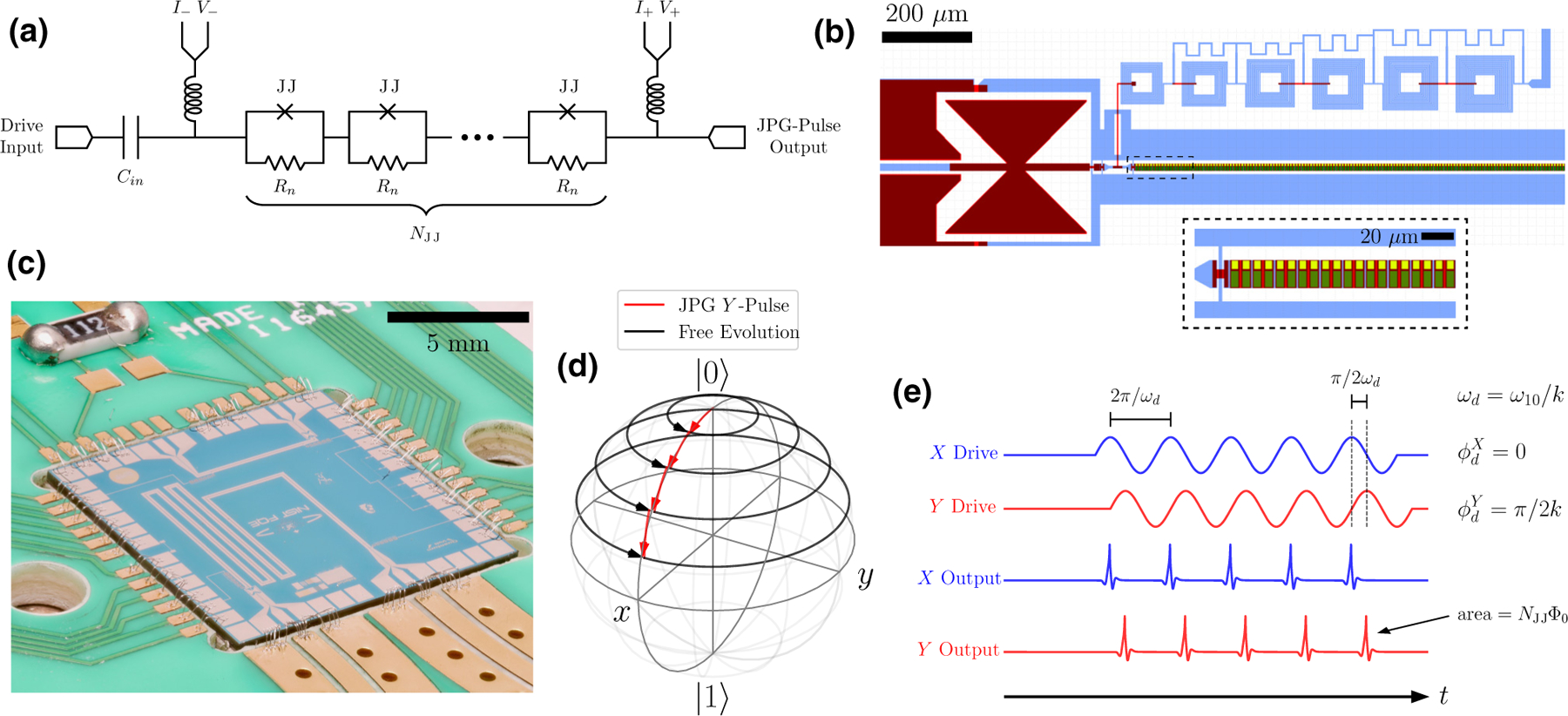
Digital qubit control with a 3-K JPG constructed using JJs with amorphous silicon (*α*Si) barriers and palladium-gold (Pd-Au) shunt resistors. (a) The circuit schematic of the JPG containing *N*_JJ_ = 4650 series JJ cells and their shunt resistors *R*_*n*_. Low-frequency inductive taps are used to provide a dc bias current *I*_*b*_ and to permit measurement of *I* -*V* curves. A dc block (*C*_in_) is used on the drive input. (b) A rendering of the input portion of the JPG circuit layout, showing approximately 250 JJs. The light blue (red) is the base (top) niobium metal layer, the green is the *α*Si JJ barrier, and the yellow is the Pd-Au shunts, *R*_*n*_. The bow-tie structure (left) forms *C*_in_ and the square-washer inductor network (top) forms the low-frequency current and voltage taps. The dashed region shows a magnified view to display the first 20 JJs and shunt resistors in the array. (c) The packaged JPG chip prior to installation in the DR: the drive input (JPG output) is the left (right) coplanar waveguide microwave launch. (d) A sketch of the qubit-state evolution on the Bloch sphere under an artificially large coupling (*δθ* = *π/*10) with *ω*_*d*_ = *ω*_10_. (e) A schematic example of a sinusoidal JPG drive and corresponding output. Under proper conditions, one JPG pulse is output per drive period, which may be done at the qubit frequency (*ω*_*d*_ = *ω*_10_) or at any subharmonic *ω*_10_/k. The orthogonal $\hat{y}$ control axis is realized by phase shifting, by $\phi_{d}^{Y}$, the drive tone with respect to the timing reference established via the $\hat{x}$ drive. The JPG emits no pulses when the drive is inactive.

The duration of this SFQ pulse is approximately *τ* = Ф_0_*/I*_*c*_*R*_*n*_, where *τ* is the JJ characteristic time, *I*_*c*_ is its critical current, and *R*_*n*_ is its normal resistance [32]. SFQ signal amplification can be achieved by connecting a series of *N*_JJ_ junctions; the pulses of which add coherently. This yields a larger pulse of area *N*_JJ_Ф_0_, which we call a *JPG pulse*. Depending on the qubit coupling to the control line, arrays with *N*_JJ_ ~ 10^2^–10^4^ are required if located at 3 K. In this work, our JPG has *N*_JJ_ = 4650, *I*_*c*_ = 3.05 mA, and *R*_*n*_ = 6.93 mΩ, resulting in a characteristic frequency of *f*_*c*_ *=* 1*/τ* = 10.2 GHz. Figures 1(a)–1(c) show, respectively, a schematic, a portion of the JPG layout, and an image of the packaged device.

If *f*_*c*_ is much larger than the qubit transition frequency, *ω*_10_/2*π* , then during pulse arrival, the qubit undergoes a discrete rotation

(2) $$\delta \theta =N_{\text{JJ}}AC_{c}\Phi_{0}\sqrt{\frac{2\omega_{10}}{\hbar C_{T}}},$$

where *A* is the JPG-qubit amplitude attenuation, *C*_*c*_ is the control-line–qubit coupling capacitance, and *C*_*T*_ is the qubit capacitance [19]. *N*_JJ_, *A*, and *C*_*c*_ may be treated as free design parameters to realize a combination of adequate control-line thermalization and tip angle per pulse, *δθ* . For three-dimensional (3D) readout-cavity configurations, *C*_*c*_ also encapsulates attenuation from pulse transit of the cavity resonance. A train of sharp pulses arriving resonantly at the qubit (*ω*_*d*_ *= ω*_10_), or at a subharmonic (*ω*_*d*_ *= ω*_10_*/k*, where *k ≥* 2 is an integer), discretely rotate the qubit around the Bloch sphere during pulse arrival, while between pulses the qubit precesses for *k* periods at fixed *θ* [see Fig. 1(d)].

In our implementation, the JPG must be driven using a sinusoidal signal at *k ≥* 2 because there is no isolation between the drive input and the device output. Otherwise, the large drive signal dominates and induces spurious qubit rotations. The generation of an integer number of JPG pulses *ℓ* is performed by sending an integer number of sinusoidal drive periods, *ν*. Under the correct bias parameters, there is a one-to-one correspondence between the number of JPG pulses generated and the number of drive periods (*ν = ℓ*). Orthogonal axis control, realized by phasing the drive signal relative to a timing reference, is depicted in Fig. 1(e). More details can be found in Sec. IV of the Supplemental Material [33].

## III. EXPERIMENTAL DETAILS

**FIG. 2.**
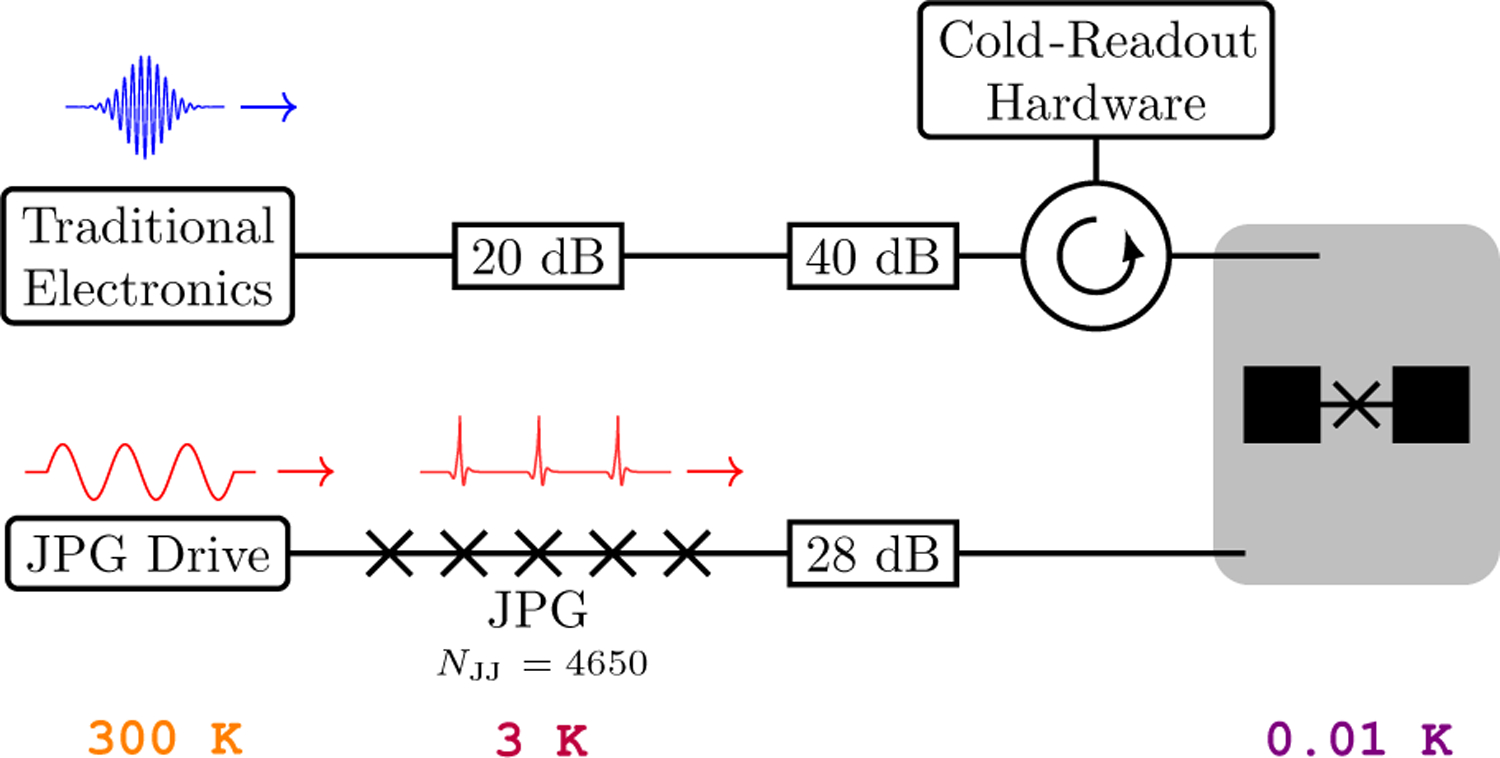
A simplified schematic of the experimental setup. JPG pulses are routed to the qubit through the weakly coupled cavity port. Sythesizers for qubit readout and conventional TSCE-based control, as well as cold-readout components [Josephson parametric amplifiers (JPAs), isolators, etc.] are attached to the strongly coupled port. JPG pulse generation is driven by a commercial 65-GSa/s arbitrary waveform generator, which is also semiconductor based, and located at ambient temperature. Control-line thermalization is achieved with attenuators or lossy microwave components at the 3-K, 1-K, 0.05-K, and 0.01-K DR temperature stages. The attenuation stacks are 20, 10, 10, and 20 dB and 9, 3, 6, and 10 dB for the TSCE and JPG control lines, respectively. For more details, see Fig. S1 in the Supplemental Material [33].

In this work, we use a transmon qubit dispersively coupled to a 3D aluminum readout cavity possessing two control lines with different coupling strengths. A simplified experimental schematic is shown in Fig. 2. The JPG is connected to the weakly coupled port of the cavity (0.175-MHz coupling rate) and the TSCE control and readout line to its strongly coupled port (2.01-MHz coupling rate). With this setup, a direct comparison of qubit performance with both control schemes is possible during the same cool-down. This qubit has been measured for a previous publication; for other parameters, see Ref. [34].

Qubit-state readout is performed by probing the qubit-state-dependent frequency shift of the cavity. The dressed cavity frequency is $\omega_{|0\rangle ,|1\rangle }=\omega_{r}\pm \chi$, where *ω*_*r*_ is the bare frequency and *χ* is the shift due to cavity-qubit coupling [35]. A Josephson parametric amplifier (JPA) [36] is operated with a gain of 20 dB (phase insensitive) to enable single-shot measurements. To minimize measurement-induced transitions, the cavity-probe tone amplitude is typically $n_{r}\approx 6≲n_{\text{crit }}/20$, where *n*_*r*_ and *n*_crit_ are the readout and critical photon numbers [37]. We perform passive qubit-state reset via relaxation over a period ≥ 15*T*_1_. The same readout procedure and instrumentation is used for both TSCE and JPG measurements.

### A. JPG operation and *X*_*π*_ calibration

After characterization with the TSCE setup, we establish operating parameters, specifically the rf drive power and dc current bias *I*_*b*_, for the JPG in which the number of output JPG pulses is equal to the number of input drive periods—called the *locking range*. Under sinusoidal rf drive at frequency *f*_*d*_, a constant voltage Shapiro step manifests at

(3) $$V=N_{\text{JJ}}\Phi_{0}f_{d}.$$

**FIG. 3.**
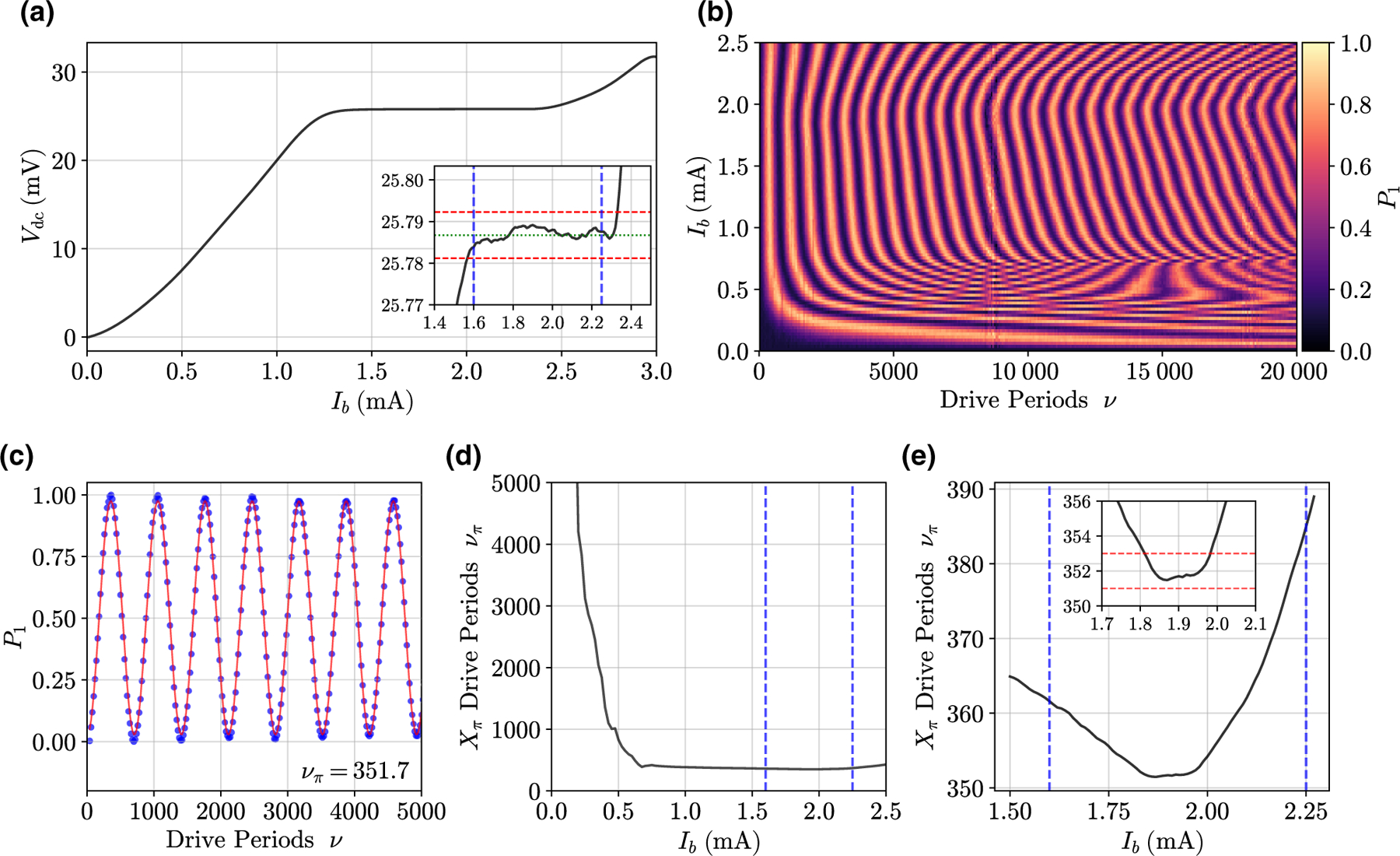
The JPG *X*_*π*_ calibration procedure. (a) JPG *I* -*V* curves—shown here with *ω*_*d*_/2*π* = 2.679 GHz—are first used to establish rough bounds on the range of *I*_*b*_, giving a constant Rabi-oscillation period with respect to the number of drive periods *ν*. The inset shows the behavior on smaller scales near the calculated Shapiro voltage [the green dotted line, given by Eq. (3)]. The red dashed lines correspond to the Shapiro voltage if there is one missing or one additional JJ. From this dc measurement, we extract a locking range of 1.6–2.25 mA, which is indicated in all plots with blue dashed lines. (b) Next, we perform a JPG Rabi-oscillation scan in which both the JPG current bias *I*_*b*_ and the number of drive periods, *ν*, are swept. (c) A single *I*_*b*_ *=* 1.9 mA JPG Rabi-oscillation measurement. A fit to the functional form sin*(*2*πν/*2*ν*_*π*_ *)* of the oscillation period yields the corresponding value for *ν*_*π*_ of 351.7 for *I*_*b*_ *=* 1.9 mA—which is rounded to 352 as we can only send an integer number of pulses. For these data, the JPG is driven at 2.685 GHz, resulting in an *X*_*π*_ gate time of *t*_gate_ = 131 ns. (d) Fitted *ν*_*π*_ at each *I*_*b*_ from data in (b), emphasizing that at low *I*_*b*_, the JPG does not pulse efficiently (or at all for *I*_*b*_ = 0). (e) The analyzed data of a finer-resolution Rabi scan around the locking range as determined in (a). The inset again shows smaller-scale features, with red dashed lines bounding *ν*_*π*_ by ±1 pulse. Here, the Rabi-oscillation period of the qubit is insensitive to variations in *I*_*b*_ over the range of 1.82–1.97 mA and *ν*_*π*_ = 352 is fixed.

When the measured voltage is constant and equal to Eq. (3) over a range of *I*_*b*_, then for any *I*_*b*_ on the Shapiro step, the device is locked. Thus, we first maximize the locking range by determining the drive power that gives the largest Shapiro steps. Figure 3(a) shows the JPG *I* -*V* curve with the maximized locking range.

For all measurements in this work, we drive at subharmonic *k* = 2 (*ω*_*d*_ = *ω*_10_/2) and use the second-harmonic power of the JPG pulse train to control the qubit. As we are restricted to subharmonic drive, *k* = 2 maximizes the locking range by making *f*_*d*_ as close as possible to *f*_*c*_ [38] and provides the highest-fidelity (fastest) gates. Next, we measure JPG-induced Rabi oscillations to characterize the JPG-qubit interaction. At the optimal drive power, we measure the number of drive periods *ν* required for a *π* rotation, *ν*_*π*_ , versus *I*_*b*_. The results of this procedure are shown in Figs. 3(b)–3(e). Fitting these Rabi oscillations at constant *I*_*b*_ yields *ν*_*π*_ *(I*_*b*_*)* and we look for regions where *ν*_*π*_ is insensitive to the number of Rabi periods (i.e., the drive time). This demonstrates locking of the JPG, where *ν = ℓ*, and a stable JPG-qubit interaction as the drive pattern is lengthened.

One may expect the entire locking range in Fig. 3(a) to give a constant *ν*_*π*_ ; however, this is not observed in Fig. 3(e). This is because the pulse width for Josephson devices operated at *f*_*d*_ *~ f*_*c*_ varies as *I*_*b*_ traverses the Shapiro step. Widening of the pulses results in a reduction of *δθ* which we discuss further in Sec. III B, and in Sec. V of the Supplemental Material [33]. Our simulations for pulse-width variation across the Shapiro step agree with previous work [39,40] and a variation of *<* 10% is expected. This restricts the region of constant *ν*_*π*_ in the Rabi measurements relative to the dc locking-range measurement. Despite these effects, Fig. 3(e) nevertheless demonstrates a range of 150 *μ*A, where *ν*_*π*_ is constant to within one pulse.

### B. Finite-Width Pulses

**FIG. 4.**
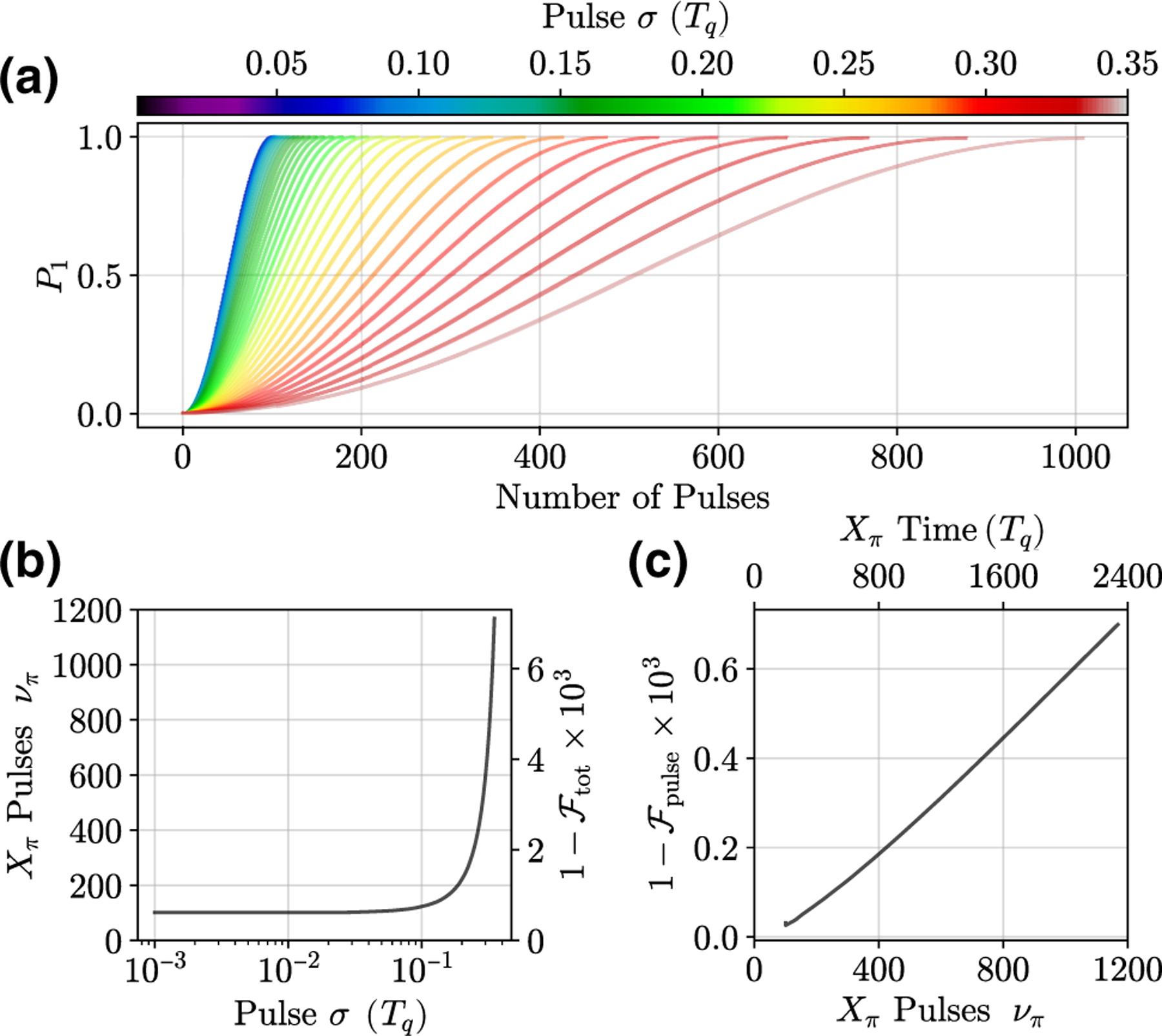
Simulations of a qubit digitally driven by a Gaussian pulse train to extract the expected *X*_*π*_ fidelity as a function of the pulse standard deviation *σ* . Here, *σ* is in units of qubit periods *T*_*q*_. Pulses are delivered at *ω*_*d*_ = *ω*_10_/2, so a pulse arrives at every other qubit period. We use energy-relaxation and dephasing rates consistent with our qubit (see Sec. V). Fits to single JPG pulses measured at room temperature yield an upper bound of *σ*_JPG_ = 0.19 *T*_*q*_ (35 ps) for the width of pulses delivered to the qubit when parametrized as a Gaussian. (a) The qubit excitation probability as a function of the number of pulses for increasing *σ* and for the first half of the first Rabi oscillation. (b) The extracted value of *ν*_*π*_ versus *σ* and the corresponding total *X*_*π*_ total infidelity, $1-{𝓕}_{\text{tot }}$. The pulse-qubit coupling is normalized such that for the Dirac-delta-function limit, *ν*_*π*_ = 100. With the coupling readjusted to match our experimental values of *ν*_*π*_ = 352 and *σ*_JPG_ = 0.19 *T*_*q*_, we obtain $1-{𝓕}_{\text{tot }}=2\times 10^{-3}$ and $1-{𝓕}_{\text{pulse }}=1\times 10^{-4}$. (c) The digital-pulse-only *X*_*π*_ infidelity, $1-{𝓕}_{\text{pulse }}$, as a function of *ν*_*π*_ —which is parametrized by *σ* in (b). $1-{𝓕}_{\text{pulse }}$ is calculated by subtracting the coherence-limit contribution to $1-{𝓕}_{\text{tot }}$.

The production of perfectly sharp pulses is not possible, so *δθ* also depends on the JPG pulse width. Indeed, we demonstrate this by broadening the JPG pulses (we heat the JPG to reduce *I*_*c*_) and observe *ν*_*π*_ to increase by approximately the same factor by which *I*_*c*_ decreases. To explore the pulse-width dependence of *δθ* , we perform simulations [41] of a qubit driven at *ω*_*d*_ *= ω*_10_/2 by Gaussian pulses the width of which (the standard deviation *σ* in units of the qubit period, *T*_*q*_) we control. Figure 4 shows our simulation results and illustrates a strongly nonlinear dependence on the qubit response for *σ >* 0.25 *T*_*q*_. For short pulses in the Dirac-delta-function limit *σ <* 0.01 *T*_*q*_, the qubit response is independent of *σ* (*ν*_*π*_ changes by less than one pulse). Section VII in the Supplemental Material [33] discusses the relationship between JJ *τ* and *σ* of a Gaussian fitted to the pulses. For our JPG with *τ* = 98 ps, the (on-chip) Gaussian-parametrized pulse width is *σ* = 17 ps.

To measure the pulse width, the JPG output is split (see Fig. S1 in the Supplemental Material [33]) and recorded with an oscilloscope at room temperature. We find that *σ* = 35 ps which, for our 5.37-GHz qubit, gives *σ* = 0.19 *T*_*q*_. This is an upper bound for the widths of pulses delivered to the qubit due to added dispersion in the additional 2 m of JPG-oscilloscope cabling compared to the JPG-qubit cable length. From the simulations (after adjusting coupling so *ν*_*π*_ = 352) and pulse-width measurements, we obtain a lower bound of our expected JPG *X*_*π*_ infidelity of 2 × 10^−3^. While the total infidelity is coherence-limit dominated, subtraction of this contribution gives the infidelity due only to the nature of control via digital pulses, $1-{𝓕}_{\text{pulse }}$. Figure 4(c) demonstrates that, for our current gate times, this digital-pulse-only infidelity is approximately 10% of the total infidelity. Furthermore, this is competitive with state-of-the-art TSCE techniques, reaching approximately 10^−4^ infidelity, and shows that there is no fundamental limitation imposed by digital control with sharp pulses [42]. For more discussion, see Sec. VII in the Supplemental Material [33].

When driven at a frequency below *f*_*c*_, the locking range of our prototype JPG decreases by a factor ∝ *f*_*d*_*/f*_*c*_ but a lower *f*_*c*_ also compromises ideal digital qubit control dynamics. More ideal control may be realized at the expense of the locking range (or vice versa) by tuning *f*_*c*_ = *I*_*c*_*R*_*n*_*/Ф*_0_—with the caveat that *σ* ≳ 0.3 *T*_*q*_ pulses are too wide for efficient digital control. We keep *σ <* 0.2 *T*_*q*_ to balance the locking range and optimal qubit dynamics. Future devices (see Sec. VI) will not possess this limitation.

## IV. COMPARISON OF QUBIT PERFORMANCE

**FIG. 5.**
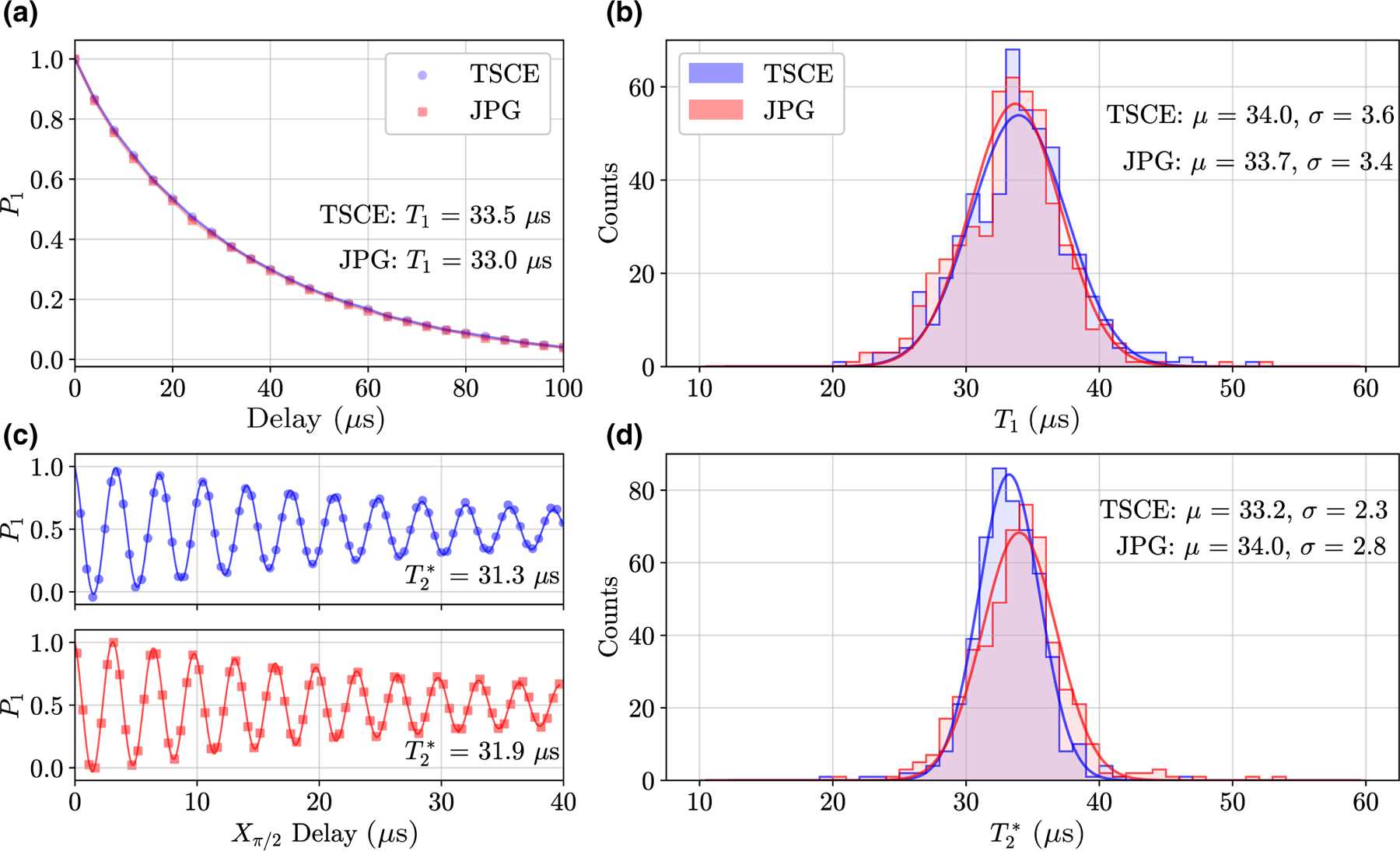
A comparison of the measured qubit lifetime *T*_1_ and the Ramsey coherence time $T_{2}^{*}$ using a TSCE setup and a JPG at 3 K. For each setup, 500 individual measurements of *T*_1_ and $T_{2}^{*}$ are taken. The data in blue (circles) are taken with the TSCE setup, while the data in red (squares) are taken with the JPG. The observed daily fluctuations in the *T*_1_ and $T_{2}^{*}$ distribution means—i.e., the uncertainty in the means—are ±0.5 *μ*s and ±2 *μ*s, respectively. The solid lines are fits to the *T*_1_ decay curves and Ramsey fringes. (a) The *T*_1_ decay curve averaged across all measurements and the fitted *T*_1_ value for each setup. (b) Histograms and Gaussian fits of the *T*_1_ distributions, showing excellent agreement in the mean (*μ*) and standard deviation (*σ* ) of the distributions. (c) The Ramsey fringe averaged across all measurements and the fitted $T_{2}^{*}$ value for each setup. (d) Histograms and Gaussian fits for $T_{2}^{*}$, again showing good agreement with each setup.

Here, we describe the side-by-side comparison of the TSCE and JPG setups through measurements of *T*_1_, $T_{2}^{*}$, and *P*_th_. For the *T*_1_ comparison, a JPG *X*_*π*_ rotation is constructed of *ν*_*π*_ *=* 352 drive periods (131-ns drive time) obtained with the calibration shown in Fig. 3. For the $T_{2}^{*}$ comparison, a JPG *X*_*π/*2_ rotation is created with a *ν*_*π*_ /2 = 176 period drive waveform. We gather statistics on 500 measurements of *T*_1_ and $T_{2}^{*}$ with each setup. The data are compiled in Fig. 5 and show energy decay curves and Ramsey fringes averaged over all measurements, as well as histograms of the extracted *T*_1_ and $T_{2}^{*}$. The small discrepancies in the distribution means are well within the expected variation in *T*_1_ and $T_{2}^{*}$ for superconducting qubits [4,28,43–46] and within the observed daily fluctuations for this device of ±0.5 *μ*s and ±2 *μ*s for the *T*_1_ and $T_{2}^{*}$ mean values, respectively. Indeed, excellent agreement is found in *T*_1_ and $T_{2}^{*}$ as measured with each setup, showing that JPG operation does not enhance relaxation or dephasing from quasiparticle poisoning or larger cavity-photon-number fluctuations.

An important validation of JPG-qubit compatibility is to demonstrate adequate thermalization when controlled with the JPG. State inversions from elevated qubit thermal occupancy can be ≳ 10% in transmon qubits with 3D aluminum readout cavities [47–49]. We define the qubit thermal occupancy *P*_th_ as the probability of incorrect state identification based on the desired preparation.

The measurement of *P*_th_ is performed in a two-part experiment [50]. First, no qubit rotation is applied and the state is simply measured. Second, we apply an *X*_*π*_ rotation to invert the qubit population and then measure. The measurements are single shot and we do not perform heralding. The total state-preparation-and-measurement (SPAM) fidelity is

(4) 𝓕=1−P(1∣0)−P(0∣1),

where *P*(*i|j* ) is the probability of measuring state |*i*⟩ when the qubit is intended to be prepared in |*j* ⟩ . Equation (4) describes the combined preparation fidelity and ability for the single-shot measurement to correctly distinguish between |0⟩ or |1⟩. Ideally, each *P*(*i|j* ) only contains contributions from thermal occupancy. In reality, both *P*(*i|j* ) include decays from correctly prepared |1⟩ and spuriously excited |0⟩ initial states and the |0⟩ and |1⟩ distribution overlap. The SPAM fidelity thus gives an upper bound on *P*_th_ and we minimize the effects of overlap infidelity and decays during measurement to improve our estimate of *P*_th_.

We limit these decays to *<* 1%, which becomes the dominant uncertainty in the *P*_th_ measurement, by shortening the readout pulse to 400 ns and we counter the corresponding reduction of the single-shot SNR using an optimal mode-matching integration weight function [50]. Overlap infidelity is minimized by increasing the cavity drive strength to separate the primary |0⟩ and |1⟩ distribution lobes. For the 400-ns readout pulse, this occurs at *n*_*r*_ *=* 50 ≈ *n*_crit_/2.3, which still avoids measurement-induced transitions [37].

**FIG. 6.**
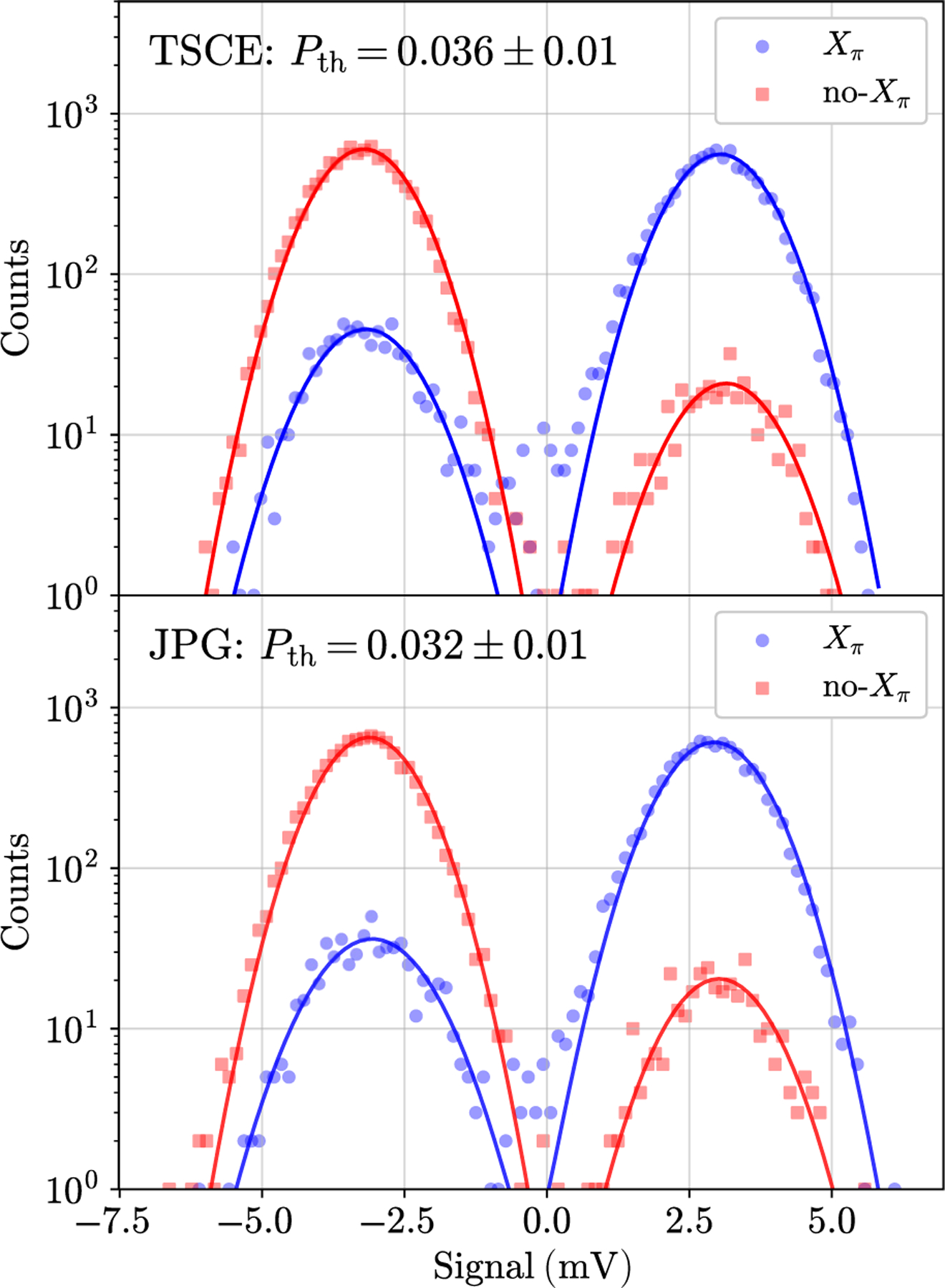
The measurement of the qubit thermal population, *P*_th_, to compare control with the TSCE (top) and JPG (bottom). The solid lines are a bimodal Gaussian fit to the data. The *P*_th_ estimate is obtained by integrating the fit to the no-*X*_*π*_ population with voltage levels greater than zero. These data points correspond to instances in which a spurious state inversion occurs, spoiling the desired |0⟩ preparation. The primary uncertainty in *P*_*th*_ using this method is due to decays during measurement, i.e., an error of approximately 1%.

Figure 6 shows data from 10^4^ measurements with each setup and bimodal Gaussian fits to the data. As the no-*X*_*π*_ case (desired preparation in |0⟩) additionally removes decays during preparation, we choose this population to bound *P*_th_ with the most accuracy. The integration of all spurious |1⟩ outcomes for this case yields excellent agreement in *P*_th_ of 0.036 ± 0.01 and 0.032 ± 0.01 for the TSCE and JPG setups, respectively. These results demonstrate that qubit thermalization is not affected when the JPG is used—our final compatibility metric of digital control using JJ-based pulses from 3 K.

## V. JPG RANDOMIZED BENCHMARKING

We now characterize JPG gate fidelities through a randomized-benchmarking (RB) routine [51–55], where we apply a sequence of *m* random gates followed by a single sequence-inverting gate. The sequence fidelity is an exponential decay

(5) $$𝓕(m)=ap^{m}+b,$$

where the constants *a* and *b* encapsulate SPAM errors and errors on the final gate. For single-qubit gates, the depolarizing parameter *p* is related to the per-gate error *r* by

(6) $$r=\frac{1}{2}(1-p).$$

The results of the same routine using the TSCE setup are provided as a reference gate error.

We choose the set of primitive and Pauli gates: {*I* , ±*X*_*π/*2_, ±*Y*_*π/*2_, ±*X*_*π*_ , ±*Y*_*π*_ }, where the idle *I* and *X*_*π*_ gate lengths are equal [56,57]. Given that *π* gates are twice as long as *π/*2 gates and the fact we use a Clifford-group subset, rescaling *m* by a factor of 1.125 permits comparison with full-Clifford-group RB [22,54]. The JPG gates are constructed as described above, while the TSCE gates use a *σ* = 35 ns Gaussian pulse truncated at ±2*σ* to closely match the JPG gate time.

**FIG. 7.**
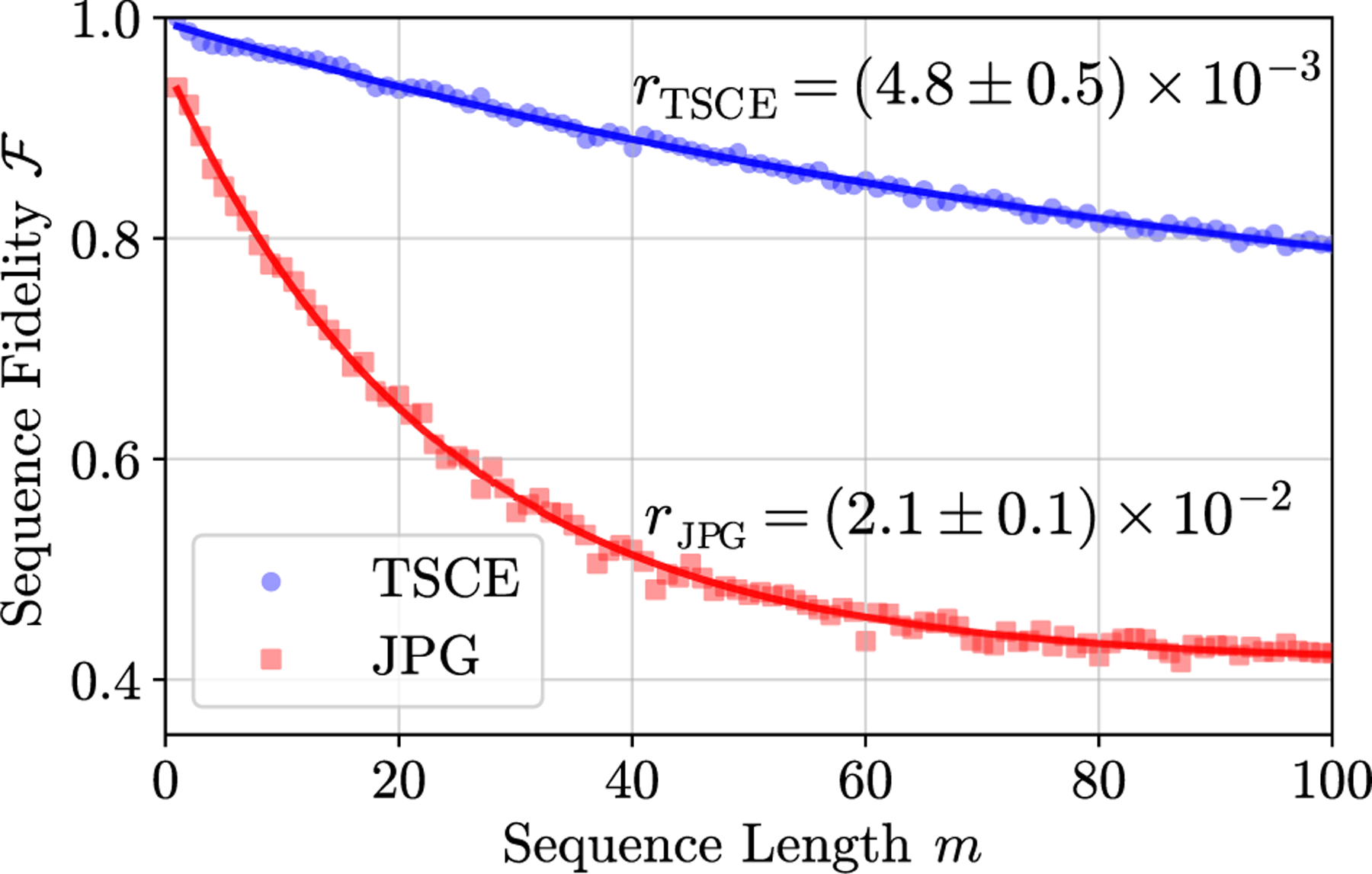
The depolarizing curve for single-qubit RB using the TSCE and 3-K JPG qubit control setups. The solid lines are a fit to Eq. (5). TSCE rotations are performed with Gaussian pulses with *σ* = 35 ns and truncated at ±2*σ* to match the JPG *X*_*π*_ gate length of 131 ns. We extract an average error per gate of *r*_TSCE_ *=* (4.8 ± 0.5) × 10^−3^ and *r*_JPG_ = (2.1 ± 0.1) × 10^−2^. The former is just over twice the coherence limit of the qubit, while the latter is a factor of 10 higher than the *X*_*π*_ fidelity of 2 × 10^−3^ as determined in simulations—which is dominated by qubit coherence. Uncertainties in *r* are determined as the standard error of the fits to Eq. (5).

In Fig. 7, we show the results of the RB routine with both setups, giving *r*_TSCE_ = (4.8 ± 0.5) × 10^−3^ and *r*_JPG_ = (2.1 ± 0.1) × 10^−2^, where the uncertainties are from the Eq. (5) fitted standard error. The JPG *r* is approximately a factor of 10 higher than the simulated single *X*_*π*_ gate error of 2.1 × 10^−3^ [58]. A detailed accounting of known possible errors (see Sec. V in the Supplemental Material [33]) from digitization, finite pulse widths, higher-state leakage, and pulse timing jitter [19,59] gives an estimated infidelity of 6 × 10^−3^. This is a factor of 3 below the RB result. We attribute the remaining error to possible systematic or coherent errors, which are presently under investigation. Regardless, these measurements serve as an excellent proof-of-concept demonstration of qubit control using 3-K JJ-based digital pulses.

## VI. SCALABILITY AND DISCUSSION OF FUTURE DEVICES

The scalability of digital qubit control using JJ devices at 3 K is promising even with the current device and configuration—which are not optimized for size or power dissipation. The JPG circuit is *<* 15 mm^2^ and the power dissipated at duty cycle *η*_*d*_ is approximately

(7) $$P=I_{c}V=\Phi_{0}N_{\text{JJ}}I_{c}f_{d}\eta_{d}.$$

For the maximum *η*_*d*_ in our experiment of 0.02, this yields *P* = 1.6 *μ*W. Commercial cryocoolers delivering approximately 1 W of cooling power at 3 K [60] permit the use of over 500 000 similar devices; occupying approximately 10^−2^ m^3^. The JPG-pulse output power is approximately −56 dBm (at *η*_*d*_ *=* 0.02), giving off-chip dissipation of the qubit drive signal of 2 nW, 0.3 nW, 0.02 nW, and 0.008 nW at the 3-K, 1-K, 0.05-K, and 0.01-K stages, respectively (for details of the JPG control-line attenuation stack, see Sec. I in the Supplemental Material [33]). Of greater consideration is dissipation of the large drive signal, which is approximately −6 dBm at the JPG input at 3 K; however, the new devices discussed below offer techniques to circumvent this limitation.

Plans for NISQ systems require approximately 10 m^3^ of cryostat volume and numerous cryocoolers. We conclude that neither the power dissipation nor the device size (the volume of silicon) present significant obstacles in scaling the number of 3-K JJ devices to control NISQ-era quantum arrays. Furthermore, our experimental and JPG architectures can both be adjusted in a straightforward manner to reduce the device size and the on-chip power dissipation, each by more than a factor of 10. Thus, the primary obstacle in scaling a 3-K JJ-based qubit control architecture, at least in the near term, is one shared by many competing qubit-control technologies: wiring and signal-routing logistics. Multichip modules [61–63], high-density and/or bandwidth interconnects [29,31], and out-of-plane coupling [64] make it feasible to overcome these challenges.

The present experiment architecture, where JPG pulses are heavily attenuated by the cavity resonance before reaching the qubit, necessitates the use of a large *N*_JJ_ to yield appropriate signal levels. This increases the device size and the power dissipation and can reduce qubit coherence by allowing the higher pulse-train harmonics to populate the cavity (not observed here). These limitations can be eliminated using two-dimensional readout-cavity qubit devices and an independent control line. With such a device, we expect to reduce the array factor to *N*_JJ_ ≈ 500 without sacrificing thermalization or the gate time.

Next-generation devices will implement an SFQ logic shift register and voltage-multiplier pulse amplification with no increase in the JJ count [16,65]. The use of a high speed clock far above the qubit spectrum eliminates the qubit-drive interaction and permits pulse delivery at *ω*_*d*_ ≥ *ω*_10_ or at variable timing. The latter has theoretically been shown to reach 99.99% fidelity with under-10-ns gates [20]. Voltage-multiplier amplification minimizes on-chip dispersion and permits narrower output pulses, enabling more ideal digital qubit dynamics. Finally, these devices permit signal routing that eliminates dissipation of the drive signal in cryogenic attenuators, which is a major consideration for the present JPG device and would strongly limit scaling of these prototype devices.

## VII. CONCLUSIONS

In this paper, we demonstrate, for the first time, successful digital control of a transmon qubit at 0.01 K using a superconducting Josephson-pulse generator located at 3 K. Through dual characterization of the system, using both TSCE [5] and the 3-K JPG, we see no reduction in intrinsic qubit performance. Specifically, we measure no negative impact on *T*_1_, $T_{2}^{*}$, or *P*_th_—indicative of the fact that quasi-particle propagation is effectively broken by locating the JJ elements and quantum circuits on separate temperature stages. Additionally, we measure an average JPG gate error of *r* = 2.1 × 10^−2^ which, considering the improvements of the future JJ devices discussed in Sec. VI are expected to reach the simulated coherence-dominated infidelity of *r*_min_ = 2 × 10^−3^.

These results enable scaled quantum information systems that leverage the merits of Josephson-based sources for qubit control: signal stability, reproducibility, SFQ pulse self-calibration, small device size, and low power dissipation. Straightforward alterations in the qubit and JPG architectures enable factors of ≥ 10 reduction in dissipation and size and future devices are expected to bring JJ-based digital gates into competition with contemporary TSCE gates. Such improvements further increase the potential value of 3-K JJ-based qubit control, as current device sizes and dissipation are commensurate with the operation of over 500 000 devices. Integration with cryoC-MOS devices [8–10] is also possible; potentially yielding a hybrid cryogenic controller that exploits the advantages of both technologies.

## Supplementary Material

supplementary material

## References

[1] Fowler AG, Mariantoni M, Martinis JM, and Cleland AN, Surface codes: Towards practical large-scale quantum computation, Phys. Rev.A 86, 032324 (2012).

[2] Kelly J, Barends R, Fowler AG, Megrant A, Jeffrey E, White TC, Sank D, Mutus JY, Campbell B, and Chen Y , State preservation by repetitive error detection in a superconducting quantum circuit, Nature 519, 66 (2015). 25739628 10.1038/nature14270

[3] Andersen CK, Remm A, Lazar S, Krinner S, Lacroix N, Norris GJ, Gabureac M, Eichler C, and Wallraff A, Repeated quantum error detection in a surface code, Nat. Phys 16, 875 (2020).

[4] Google Quantum AI, Exponential suppression of bit or phase errors with cyclic error correction, Nature 595, 383 (2021). 34262210 10.1038/s41586-021-03588-y PMC8279951

[5] Krantz P, Kjaergaard M, Yan F, Orlando TP, Gustavsson S, and Oliver WD, A quantum engineer’s guide to superconducting qubits, Appl. Phys. Rev 6, 021318 (2019).

[6] Krinner S, Storz S, Kurpiers P, Magnard P, Heinsoo J, Keller R, Luetolf J, Eichler C, and Wallraff A, Engineering cryogenic setups for 100-qubit scale superconducting circuit systems, EPJ Quantum Technol 6, 2 (2019).

[7] van Dijk J, Kawakami E, Schouten R, Veldhorst M, Vandersypen L, Babaie M, Charbon E, and Sebastiano F, Impact of Classical Control Electronics on Qubit Fidelity, Phys. Rev. Appl 12, 044054 (2019).

[8] Van Dijk JPG, Patra B, Subramanian S, Xue X, Samkharadze N, Corna A, Jeon C, Sheikh F, Juarez-Hernandez E, Esparza BP, Rampurawala H, Carlton BR, Ravikumar S, Nieva C, and Kim S , A scalable cryo-CMOS controller for the wideband frequency-multiplexed control of spin qubits and transmons, IEEE J. Solid-State Circuits 55, 2930 (2020).

[9] Bardin JC, Jeffrey E, Lucero E, Huang T, Das S, Sank DT, Naaman O, Megrant AE, Barends R, White T, Giustina M, Satzinger KJ, Arya K, Roushan P, and Chiaro B , Design and characterization of a 28-nm bulk-CMOS cryogenic quantum controller dissipating less than 2 mW at3 K, IEEE J. Solid-State Circuits 54, 3043 (2019).

[10] Pauka S, Das K, Kalra R, Moini A, Yang Y, Trainer M, Bousquet A, Cantaloube C, Dick N, and Gardner G , A cryogenic CMOS chip for generating control signals for multiple qubits, Nat. Electron 4, 64 (2021).

[11] Sirois AJ, Castellanos-Beltran M, Fox AE, Benz SP, and Hopkins PF, Josephson microwave sources applied to quantum information systems, IEEE Trans. Quantum Eng 1, 1 (2020).

[12] Ball H, Oliver WD, and Biercuk MJ, The role of master clock stability in quantum information processing, Npj Quantum Inf 2, 1 (2016).

[13] Rüfenacht A, Howe LA, Fox AE, Schwall RE, Dresselhaus PD, Burroughs CJ, Benz SP, and Benz SP, Cryocooled 10 V programmable Josephson voltage standard, IEEE. Trans. Instrum. Meas 64, 1477 (2015).

[14] Burroughs CJ, Dresselhaus PD, Rufenacht A, Olaya D, Elsbury MM, Tang Y-H, and Benz SP, NIST 10 V programmable Josephson voltage standard system, IEEE. Trans. Instrum. Meas 60, 2482 (2011).

[15] Brevik JA, Donnelly CA, Flowers-Jacobs NE, Fox AE, Hopkins PF, Dresselhaus PD, and Benz SP, Radio-Frequency Waveform Synthesis with the Josephson Arbitrary Waveform Synthesizer, 2018 Conference on Precision Electromagnetic Measurements, 1 (2018).

[16] Hopkins PF, Brevik JA, Castellanos-Beltran M, Donnelly CA, Flowers-Jacobs NE, Fox AE, Olaya D, Dresselhaus PD, and Benz SP, RF waveform synthesizers with quantum-based voltage accuracy for communications metrology, IEEE Trans. Appl. Supercond 29, 1 (2019).

[17] Rüfenacht A, Flowers-Jacobs NE, and Benz SP, Impact of the latest generation of Josephson voltage standards in ac and dc electric metrology, Metrologia 55, S152 (2018).

[18] Fox AE, Dresselhaus PD, Rüfenacht A, Sanders A, and Benz SP, Junction yield analysis for 10 V programmable Josephson voltage standard devices, IEEE Trans. Appl. Supercond 25, 1 (2015).32863691

[19] McDermott R and Vavilov MG, Accurate Qubit Control with Single Flux Quantum Pulses, Phys. Rev. Appl 2, 014007 (2014).

[20] Liebermann PJ and Wilhelm FK, Optimal Qubit Control Using Single-Flux Quantum Pulses, Phys. Rev. Appl 6, 024022 (2016).

[21] McDermott R, Vavilov MG, Plourde BLT, Wilhelm FK, Liebermann PJ, Mukhanov OA, and Ohki TA, Quantum-classical interface based on single flux quantum digital logic, Quantum Sci. Technol 3, 024004 (2018).

[22] Leonard E, Beck MA, Nelson J, Christensen B, Thorbeck T, Howington C, Opremcak A, Pechenezhskiy I, Dodge K, Dupuis N, Hutchings M, Ku J, Schlenker F, Suttle J, and Wilen C , Digital Coherent Control of a Superconducting Qubit, Phys. Rev. Appl 11, 014009 (2019).

[23] Patel U, Pechenezhskiy IV, Plourde BLT, Vavilov MG, and McDermott R, Phonon-mediated quasiparticle poisoning of superconducting microwave resonators, Phys. Rev. B 96, 220501 (2017).

[24] Martinis JM, Ansmann M, and Aumentado J, Energy Decay in Superconducting Josephson-Junction Qubits from Nonequilibrium Quasiparticle Excitations, Phys. Rev. Lett 103, 097002 (2009). 19792820 10.1103/PhysRevLett.103.097002

[25] Ballard A, Iaia V, McBroom T, Liu Y, Dodge K, Ku J, Liu C-H, Opremcak A, Wilen C, and Leonard E , Single Flux Quantum-Based Superconducting Qubit Control and Quasiparticle Mitigation: Part I, Bulletin of the American Physical Society (2021).

[26] Liu C, Opremcak A, Wilen C, Leonard E, Beck M, Abdullah S, Ballard A, Iaia V, McBroom T, and Liu Y , Single Flux Quantum-Based Superconducting Qubit Control and Quasiparticle Mitigation: Part 2, Bulletin of the American Physical Society (2021).

[27] Hosseinkhani A, Riwar R-P, Schoelkopf RJ, Glazman LI, and Catelani G, Optimal Configurations for Normal-Metal Traps in Transmon Qubits, Phys. Rev. Appl 8, 064028 (2017).

[28] Martinis JM, Saving superconducting quantum processors from decay and correlated errors generated by gamma and cosmic rays, Npj Quantum Inf 7, 1 (2021).

[29] Smith JP, Mazin BA, Walter AB, Daal M, Bailey J III , Bockstiegel C, Zobrist N, Swimmer N, Steiger S, and Fruitwala N, Flexible coaxial ribbon cable for high-density superconducting microwave device arrays, IEEE Trans. Appl. Supercond 31, 1 (2020).

[30] Walter AB, Bockstiegel C, Mazin BA, and Daal M, Laminated NbTi-on-kapton microstrip cables for flexible sub-kelvin rf electronics, IEEE Trans. Appl. Supercond 28, 1 (2018).

[31] Tuckerman DB, Hamilton MC, Reilly DJ, Bai R, Hernandez GA, Hornibrook JM, Sellers JA, and Ellis CD, Flexible superconducting Nb transmission lines on thin film polyimide for quantum computing applications, Supercond. Sci. Technol 29, 084007 (2016).

[32] Tinkham M, Introduction to Superconductivity (Dover Publications Inc., Minneola, New York, 1996).

[33] See the Supplemental Material at 10.1103/PRXQuantum.3.010350 for information regarding the full experimental schematic, the Rabi and Ramsey JPG scans, and JPG details.

[34] Lecocq F, Quinlan F, Cicak K, Aumentado J, Diddams S, and Teufel J, Control and readout of a super-conducting qubit using a photonic link, Nature 591, 575 (2021). 33762768 10.1038/s41586-021-03268-x

[35] Bianchetti R, Filipp S, Baur M, Fink JM, Göppl M, Leek PJ, Steffen L, Blais A, and Wallraff A, Dynamics of dispersive single-qubit readout in circuit quantum electrodynamics, Phys. Rev.A 80, 043840 (2009).

[36] Castellanos-Beltran M and Lehnert K, Widely tunable parametric amplifier based on a superconducting quantum interference device array resonator, Appl. Phys. Lett 91, 083509 (2007).

[37] Sank D, Chen Z, Khezri M, Kelly J, Barends R, Campbell B, Chen Y, Chiaro B, Dunsworth A, Fowler A, Jeffrey E, Lucero E, Megrant A, Mutus J, and Neeley M , Measurement-Induced State Transitions in a Super-conducting Qubit: Beyond the Rotating Wave Approximation, Phys. Rev. Lett 117, 190503 (2016). 27858439 10.1103/PhysRevLett.117.190503

[38] Benz SP and Hamilton CA, A pulse-driven programmable Josephson voltage standard, Appl. Phys. Lett 68, 3171 (1996).

[39] C. A. Donnelly, N. E. Flowers-Jacobs, J. A. Brevik, A. E. Fox, P. D. Dresselhaus, P. F. Hopkins, and S. P. Benz, 1 GHz waveform synthesis with Josephson junction arrays, IEEE Trans. Appl. Supercond 30, 1 (2020). 10.1109/TASC.2019.2929481 PMC6774383 31579273

[40] Babenko AA, Boaventura AS, Flowers-Jacobs NE, Brevik JA, Fox AE, Williams DF, Popović Z, Dresselhaus PD, and Benz SP, in 2020 IEEE/MTT-S International Microwave Symposium (IMS) (2020), p. 936.

[41] Johansson JR, Nation PD, and Nori F, QuTiP: An open-source PYTHON framework for the dynamics of open quantum systems, Comput. Phys. Commun 183, 1760 (2012).

[42] These simulations are repeated using simulated SFQ pulses with the JPG τ = 98 ps (see the Supplemental Material [33]) and show no significant change in fidelity.

[43] Burnett JJ, Bengtsson A, Scigliuzzo M, Niepce D, Kudra M, Delsing P, and Bylander J, Decoherence bench-marking of superconducting qubits, Npj Quantum Inf 5, 1 (2019).

[44] Vepsäläinen AP, Karamlou AH, Orrell JL, Dogra AS, Loer B, Vasconcelos F, Kim DK, Melville AJ, Niedzielski BM, and Yoder JL , Impact of ionizing radiation on superconducting qubit coherence, Nature 584, 551 (2020). 32848227 10.1038/s41586-020-2619-8

[45] McRae CRH, Wang H, Gao J, Vissers MR, Brecht T, Dunsworth A, Pappas DP, and Mutus J, Materials loss measurements using superconducting microwave resonators, Rev. Sci. Instrum 91, 091101 (2020). 33003823 10.1063/5.0017378

[46] McEwen M, Faoro L, Arya K, Dunsworth A, Huang T, Kim S, Burkett B, Fowler A, Arute F, and Bardin al JC, Resolving catastrophic error bursts from cosmic rays in large arrays of superconducting qubits, arXiv preprint arXiv:2104.05219 (2021).

[47] Wenner J, Yin Y, Lucero E, Barends R, Chen Y, Chiaro B, Kelly J, Lenander M, Mariantoni M, Megrant A, Neill C, O’Malley PJJ, Sank D, Vainsencher A, and Wang H , Excitation of Superconducting Qubits from Hot Nonequilibrium Quasiparticles, Phys. Rev. Lett 110, 150502 (2013). 25167235 10.1103/PhysRevLett.110.150502

[48] Jin XY, Kamal A, Sears AP, Gudmundsen T, Hover D, Miloshi J, Slattery R, Yan F, Yoder J, Orlando TP, Gustavsson S, and Oliver WD, Thermal and Residual Excited-State Population in a 3D Transmon Qubit, Phys. Rev. Lett 114, 240501 (2015). 26196969 10.1103/PhysRevLett.114.240501

[49] Jeffrey E, Sank D, Mutus JY, White TC, Kelly J, Barends R, Chen Y, Chen Z, Chiaro B, Dunsworth A, Megrant A, O’Malley PJJ, Neill C, Roushan P, and Vainsencher A , Fast Accurate State Measurement with Superconducting Qubits, Phys. Rev. Lett 112, 190504 (2014). 24877923 10.1103/PhysRevLett.112.190504

[50] Walter T, Kurpiers P, Gasparinetti S, Magnard P, Potočnik A, Salathé Y, Pechal M, Mondal M, Oppliger M, Eichler C, and Wallraff A, Rapid High-Fidelity Single-Shot Dispersive Readout of Superconducting Qubits, Phys. Rev. Appl 7, 054020 (2017).

[51] Knill E, Leibfried D, Reichle R, Britton J, Blakestad RB, Jost JD, Langer C, Ozeri R, Seidelin S, and Wineland DJ, Randomized benchmarking of quantum gates, Phys. Rev. A 77, 012307 (2008).

[52] Magesan E, Gambetta JM, Johnson BR, Ryan CA, Chow JM, Merkel ST, da Silva MP, Keefe GA, Rothwell MB, Ohki TA, Ketchen MB, and Steffen M, Efficient Measurement of Quantum Gate Error by Interleaved Randomized Benchmarking, Phys. Rev. Lett 109, 080505 (2012). 23002731 10.1103/PhysRevLett.109.080505

[53] Magesan E, Gambetta JM, and Emerson J, Scalable and Robust Randomized Benchmarking of Quantum Processes, Phys. Rev. Lett 106, 180504 (2011). 21635076 10.1103/PhysRevLett.106.180504

[54] Chen Z, Ph.D. thesis, University of California Santa Barbara, 2018.

[55] Rol MA, Bultink CC, O’Brien TE, de Jong SR, Theis LS, Fu X, Luthi F, Vermeulen RFL, de Sterke JC, Bruno A, Deurloo D, Schouten RN, Wilhelm FK, and DiCarlo L, Restless Tuneup of High-Fidelity Qubit Gates, Phys. Rev. Appl 7, 041001 (2017).

[56] Petit L, Eenink H, Russ M, Lawrie W, Hendrickx N, Philips S, Clarke J, Vandersypen L, and Veldhorst M, Universal quantum logic in hot silicon qubits, Nature 580, 355 (2020). 32296188 10.1038/s41586-020-2170-7

[57] McKay DC, Sheldon S, Smolin JA, Chow JM, and Gambetta JM, Three-Qubit Randomized Benchmarking, Phys. Rev. Lett 122, 200502 (2019). 31172740 10.1103/PhysRevLett.122.200502

[58] Note that a small measurement error is present in all the JPG RB data, which are taken first and on a separate cool-down than the TSCE RB. This error is remedied before the TSCE measurement is performed and is responsible for the offset in the JPG curve (Fig. 7). We emphasize that this has no influence on the extracted r for either setup.

[59] Rylyakov AV and Likharev KK, Pulse jitter and timing errors in RSFQ circuits, IEEE Trans. Appl. Supercond 9, 3539 (1999).

[60] Tsan T, Galitzki N, Ali AM, Arnold K, Coppi G, Ervin T, Foote L, Keating B, Lashner J, Orlowski-Scherer J, Randall MJ, Seibert J, Spisak J, Teply GP, and Xu Z , The effects of inclination on a two stage pulse tube cryocooler for use with a ground based observatory, Cryogenics 117, 103323 (2021).

[61] Holman N, Rosenberg D, Yost D, Yoder J, Das R, Oliver WD, McDermott R, and Eriksson M, 3D integration and measurement of a semiconductor double quantum dot with a high-impedance TiN resonator, Npj Quantum Inf 7, 1 (2021).

[62] Gold A, Paquette J, Stockklauser A, Reagor MJ, Alam MS, Bestwick A, Didier N, Nersisyan A, Oruc F, and Razavi A , Entanglement across separate silicon dies in a modular superconducting qubit device, Npj Quantum Inf 7, 1 (2021).

[63] Hidaka M, Japanese Activities for Superconducting Circuits Using Flip-Chip Configurations, IEEE CSC and ESAS Superconductivity News Forum (2019).

[64] Rahamim J, Behrle T, Peterer M, Patterson A, Spring P, Tsunoda T, Manenti R, Tancredi G, and Leek P, Double-sided coaxial circuit QED with out-of-plane wiring, Appl. Phys. Lett 110, 222602 (2017).

[65] Castellanos-Beltran MA, Olaya DI, Sirois AJ, Donnelly CA, Dresselhaus PD, Benz S, and Hopkins PF, Single-flux-quantum multiplier circuits for synthesizing gigahertz waveforms with quantum-based accuracy, IEEE Trans. Appl. Supercond 31, 1 (2021). 10.1109/tasc.2021.3057013 PMC11558795 39540146

