# Supplementary material for: Digital Control of a Superconducting Qubit Using a Josephson Pulse Generator at 3 K
Source: PRX quantum. Author manuscript; Available in PMC 2023 Jan 31. (PMC9888300; doi:10.1103/prxquantum.3.010350)
Supplement: supplementary material [file NIHMS1858172-supplement-supplementary_material.pdf]

Supplementary Material: Digital control of a superconducting qubit using a Josephson pulse generator at 3 K

I. FULL EXPERIMENTAL SCHEMATIC

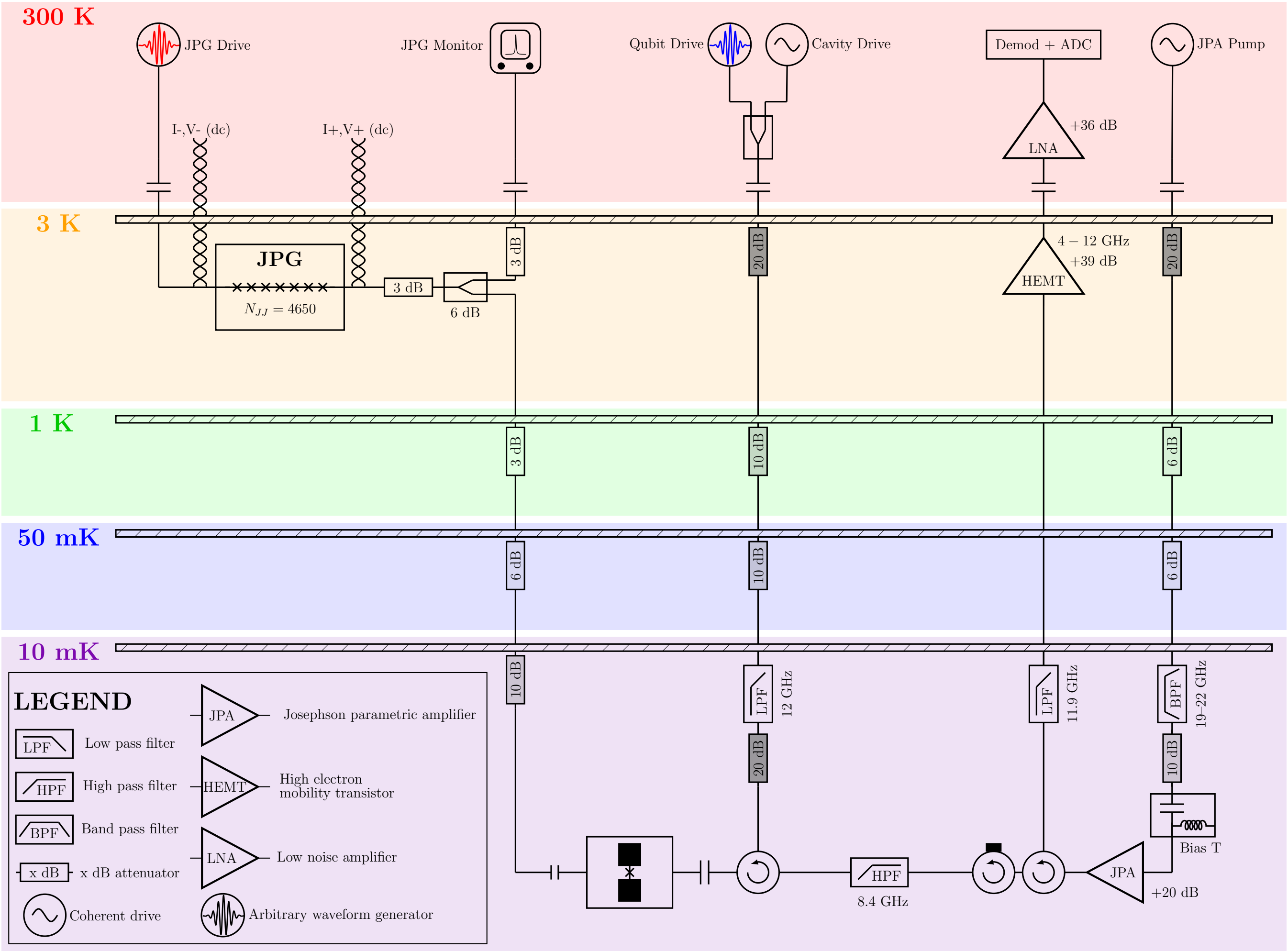

FIG. S1. (Color) Detailed schematic of the setup used to evaluate qubit control using a Josephson Pulse Generator (JPG) at 3 K in a commercial DR. The dc connection to the bias T (via twisted pair) is used to provide a dc flux bias to the JPA. Both the JPA dc flux bias and the I and V JPG taps are low-pass filtered at 1.9 MHz and the JPG taps are further filtered with an on-chip inductor network (see Fig 1(b) in the main body). We insert an attenuator between the JPG splitter output and the 3 K plate to minimize reflections from signals exiting the cryostat and prevent them from affecting the rf JPG signal on-chip.

We present the full experimental diagram in Fig. S1. The JPG has two sets of twisted pair leads connected to on-chip inductive taps to permit low-frequency I-V characterization of the JPG. A splitter is also used on the JPG rf output line for analysis of high frequency characteristics using ambient instrumentation. A 2.85 GHz low pass filter with over 60 dB insertion loss at 5 GHz is used at 300 K to fully prevent any higher harmonic content of the JPG clock signal from driving the qubit.

II. RABI AND RAMSEY FREQUENCY SWEEPS

In Fig. S2 we present generalized Rabi and Ramsey scans where we sweep the JPG drive frequency $\omega_d + \delta\omega_d = \omega_{10}/2$. This is simply a demonstration that we may make connection with various routines and protocols with the JPG that are common with TSCE control setups.

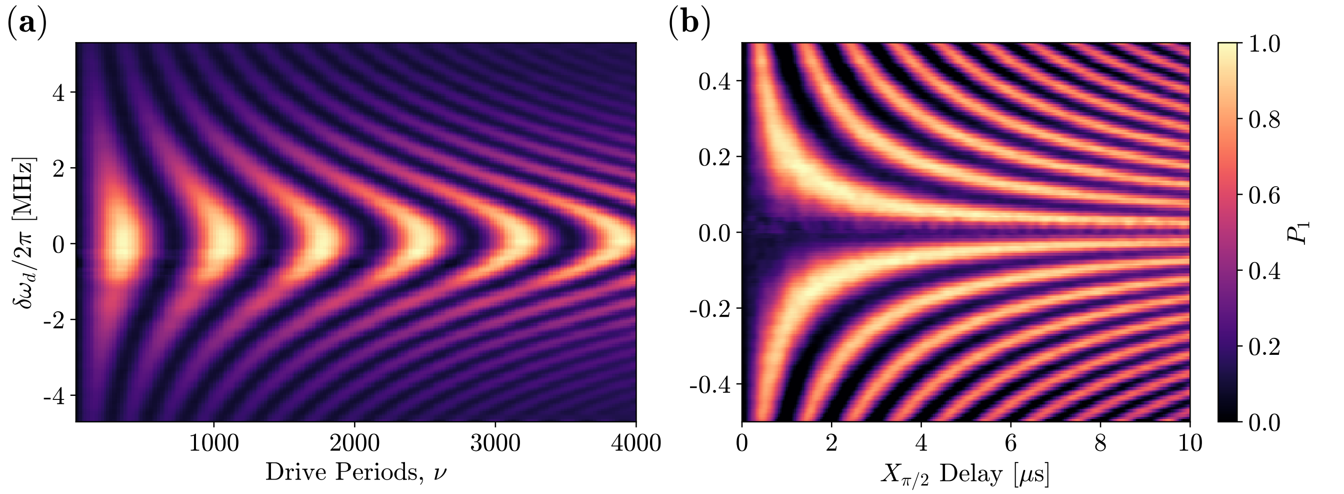

FIG. S2. (Color) **(a)** JPG Rabi chevron at fixed I_b. **(b)** Generalized Ramsey scan. In both measurements the JPG drive frequency ω_d is swept in a small region $\delta\omega_d = \omega_d - \omega_{10}/2$.

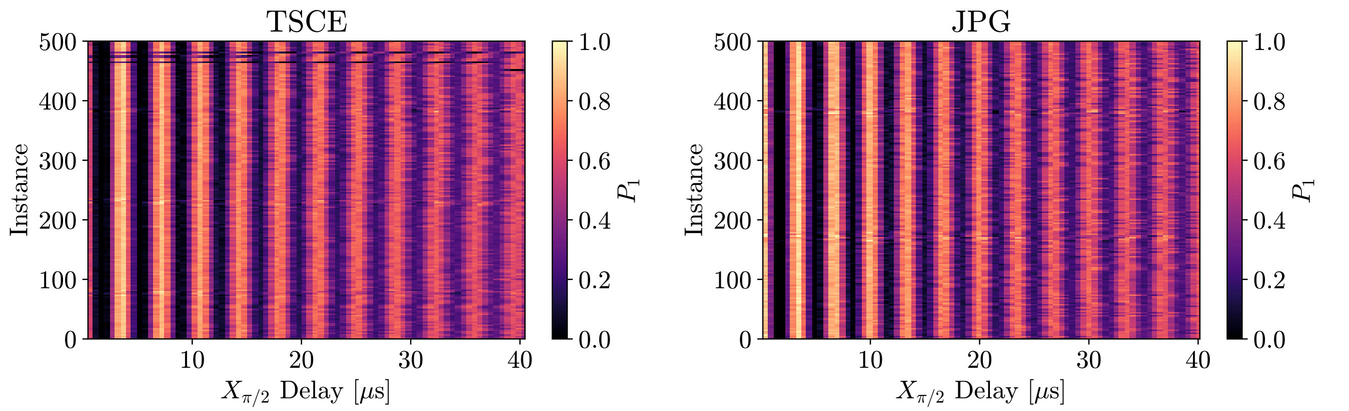

FIG. S3. (Color) Compilation colorplot of all Ramsey fringes gathered with both control setups for statistical evaluation of the insensitivity of the qubit coherence time to the control setup used. The three dark, horizontal, stripe-like features in the TSCE compilation are due to instances where the pulse-shaping AWG loses locking during the course of the measurement of a single fringe.

III. RAMSEY FRINGE COMPILATION

In Fig. S3 we compile all Ramsey fringes gathered from the T_2^* measurements in Sec. IV. The consistent level of fluctuations in the fringe frequency indicate a similar level of stability in ω_{10} with both qubit control setups.

IV. ORTHOGONAL AXIS CONTROL AND CONSTRUCTION OF JPG DRIVE WAVEFORMS

Here we provide details of JPG drive waveforms and the procedure to establish and verify orthogonal axis control of the qubit. For orthogonal control, first a timing reference is established to define the $\hat{x}$ control axis ($\phi_d^{\hat{x}} = 0$). At subharmonic drive $k \geq 2$ the $\hat{y}$ control axis is realized by phasing the drive signal to allow the qubit to precess by $\phi_q = \pi/2$ before the control pulse train arrives [22]. Intuitively, there are k qubit periods per drive period and during each qubit period the qubit phase advances relative to the drive by a factor $2\pi k$. Therefore, to accumulate the required $\phi_q = \pi/2$ in a single drive period, the $\hat{y}$ drive signal must be phased by $\phi_d^{\hat{y}} = \pi/2k$ relative to the $\hat{x}$ drive timing reference.

Drive waveforms are constructed from sine waves with an integer number of samples per sine period with the integer chosen to be compatible with the DAC sample rate of the AWG and also results in synthesis at the proper

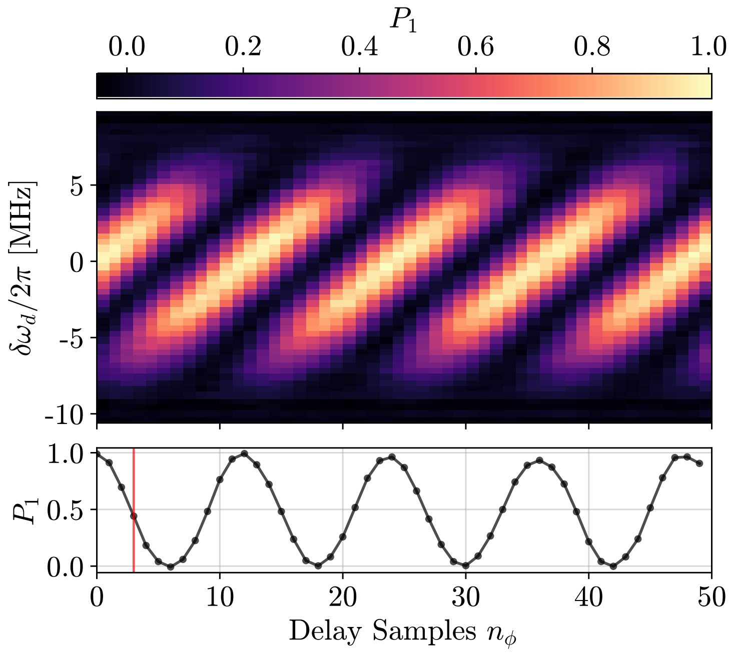

FIG. S4. (Color) Calibration of the delay samples n_ϕ, with respect to the $\hat{x}$ timing reference, which manifests the orthogonal $\hat{y}$ control. In this measurement the clock frequency and number of zero delay samples n_ϕ between sine patterns of $\nu_\pi/2$ periods are swept. When P_1 progresses from 1 (at $n_\phi = 0$) to 0.5 we have realized an $X_{\pi/2}$+$Y_{\pi/2}$ gate sequence; determining the correct value for n_ϕ for $\hat{y}$ control. Using sine patterns with 24 samples per period and with $\omega_d = \omega_{10}/2$ we expect proper phasing to occur at $n_\phi = 3$ ($\phi_d = \pi/4$). The black trace (horizontal slice at $\delta\omega_d = 0$) and vertical red line demonstrate correct calibration of n_ϕ.

drive frequency. For all qubit control experiments we generate JPG pulses at the qubit subharmonic $k = 2$ and use sine periods of length 24 samples, leading to an AWG clock frequency of 64.32 GSa/s to synthesize 2.68 GHz clocking sine pulses. For $\omega_d = \omega_{10}/2$ the expected delay required for $\hat{y}$ rotations is $\phi_d^y = \pi/4$.

Phasing between the timing reference (in-phase $\hat{x}$ drive) and the $\hat{y}$ control axis is verified by performing a Ramsey-like experiment. The JPG clock frequency is swept, as well as the delay between two "$X_{\pi/2}$" pulses. I.e. we begin with zero delay, yielding an X_π pulse, and increase the delay by inserting a number n_ϕ of zero delay samples until the rotation sequence $X_{\pi/2} + Y_{\pi/2}$ is observed. Thus the signature of the $\hat{y}$ axis calibration experiment will be a measurement of the qubit in the $|1\rangle$ state ($P_1 = 1$) and decreasing to an equal $|0\rangle + |1\rangle$ ($P_1 = 0.5$) superposition when the proper $\hat{y}$ phase (n_ϕ) is reached. Oscillations then proceed with a period of $4n_\phi$ delay samples as the required delay in the qubit frame is one-fourth of a period. An example of this calibration routine in shown in Fig. S4.

For the JPG RB tests we note that there are often subsets of the full pattern resembling $X_\pi + Y_\pi$, or $Y_\pi + X_\pi$ gate combinations. Referring to Fig. 1(d), we see that to construct a $\hat{y}$ rotation, the phase of the drive must be delayed relative to the initial $\hat{x}$ timing reference by $\phi_d^y = \pi/2k$. Similarly, to achieve an $\hat{x}$ rotation immediately following a $\hat{y}$ rotation, the drive phase must be advanced by the same amount to re-establish the null timing reference phase. To this end, every X_π and Y_π JPG waveform is $\nu_\pi + 2$ periods in length. There are ν_π periods of active drive, with the additional two periods of idle divided appropriately at the beginning and end of the waveform to permit delaying and advancing the drive phase as needed. The same procedure is followed for $X_{\pi/2}$ and $Y_{\pi/2}$ rotations. Due to the unidirectional nature of digital qubit control using a JPG, $-\pi/2$ rotations are achieved using $\phi_d^{-x} = 3\pi/2$ and $\phi_d^{-Y} = \pi/2n + 3\pi/2$ phases relative to the timing reference (we take $X_\pi = -X_\pi$ and $Y_\pi = -Y_\pi$ as the ideal final states are identical to those from positive gates). Note that, for both positive and negative $\pi/2$ rotations, the drive signals are equal in length as described above.

V. DIGITAL QUBIT CONTROL FIDELITY LIMITS

Multiple factors arise in a digital qubit control architecture which affect fidelity and are not present in systems using TSCE-based microwave pulses. They are: digitization error, error due to the finite temporal width of the digital

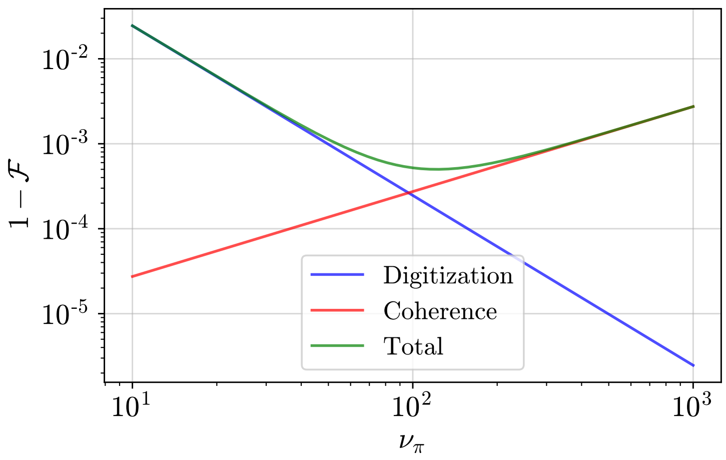

FIG. S5. (Color) JPG X_π digitization infidelity as a function of ν_π, the number of pulses, and thus the gate time, required for an X_π rotation. For small ν_π (i.e. fast gates) the digitization error from the finite single pulse rotation angle $\delta\theta$ dominates, whereas for large ν_π the qubit coherence limit becomes dominant. We use representative values for our qubit of $T_1 = T_2^* = 34\ \mu$s (i.e. $T_\phi = 68\ \mu$s). The qubit frequency is $\omega_{10}/2\pi = 5.37$ GHz and drive at $\omega_d = \omega_{10}/2$.

pulses, higher state leakage of the pulse train spectrum due to finite qubit anharmonicity, and pulse arrival timing jitter.

Digitization error arises from eliminating the continuous variable corresponding to the X_π pulse power and manifests as a maximal over- or under-rotation by (at most) $\pm 1/2$ pulse. The digitization error is

$$1 - \mathcal{F}_{dig} = 1 - \sin^2\left(\pi \frac{\nu_\pi \pm 1/2}{\nu_\pi}\right), \tag{S1}$$

which decreases as ν_π increases. With finite qubit coherence time a sweet spot arises which minimizes the infidelity, as is shown in Fig. S5. Note that as the qubit lifetime increases the value of ν_π which minimizes the combined digitization and coherence-limited infidelity also increases. For $\nu_\pi = 352$ the digitization infidelity of 3×10^{-5} is much smaller than the infidelity due to qubit coherence.

The second affect on the gate fidelity is due to the finite temporal width of the control pulses. As discussed in the main text, Sec. III B, we simulate these effects [40] using a train of Gaussian pulses delivered at $\omega_d = \omega_{10}/2$ to evolve the qubit state and study the fidelity of X_π rotations as a function of the pulse standard deviation σ. The Hamiltonian for the system is

$$\hat{H} = \hat{H}_0 + \hat{H}_d = \frac{1}{2}\hat{\sigma}_z - \Omega_d s_d(t)\hat{\sigma}_x \tag{S2}$$

where Ω_d and $s_d(t)$ describe the coupling strength and the pulse train time-dependent amplitude, respectively. Note that due to the pulse drive's time-dependent nature we cannot make a rotating wave approximation. Since our simulations use a true two-level system, there is no leakage outside of $\{|0\rangle, |1\rangle\}$ so P_1 can be used to calculate the X_π fidelity $\mathcal{F}$. To determine $\mathcal{F}$ we evaluate P_1 only at the center of the idle qubit period following a pulse and take the resultant maximum value as $\mathcal{F}$. This also defines the value of $\nu_\pi(\sigma)$, i.e. the number of pulses which maximize the following idle qubit period P_1 as a function of σ. As σ is increased, the pulse train power at the second harmonic, and thus the tip angle per pulse, $\delta\theta$, diminishes so more pulses are required to realize a π rotation with wider pulses (this is in conjunction with distributed pulse energy delivery as the qubit precesses, discussed below). In turn, $\nu_\pi(\sigma)$ increases so we must recalculate $\nu_\pi(\sigma)$ at each pulse width. For these simulations we include loss with realistic values of $T_1 = T_2^* = 34\ \mu$s ($T_\phi = 68\ \mu$s) for our qubit. Fig. 4 in the main body shows the X_π Rabi oscillation curves, $\nu_\pi(\sigma)$, and the corresponding $X_\pi(\sigma)$ fidelity.

If the Gaussian pulse duration ($\sim 4\sigma$) approaches $2\pi/\omega_{10}$, the pulses no longer deliver the majority of their power when the qubit is aligned with the desired control axes. Rather, the pulses turn on and off slowly relative to the qubit period and their action is distributed over a non-negligible portion of the qubit precession. For a wide pulse near its peak, the pulse delivers the majority of its energy and rotates the qubit state $|\psi\rangle$ along the target control axis. However, the long tails before and after the peak impart lesser rotations while the qubit advances phase at ω_{10}. As the qubit precesses, $|\psi\rangle$ moves through having a component anti-aligned with the target control axis, to being aligned,

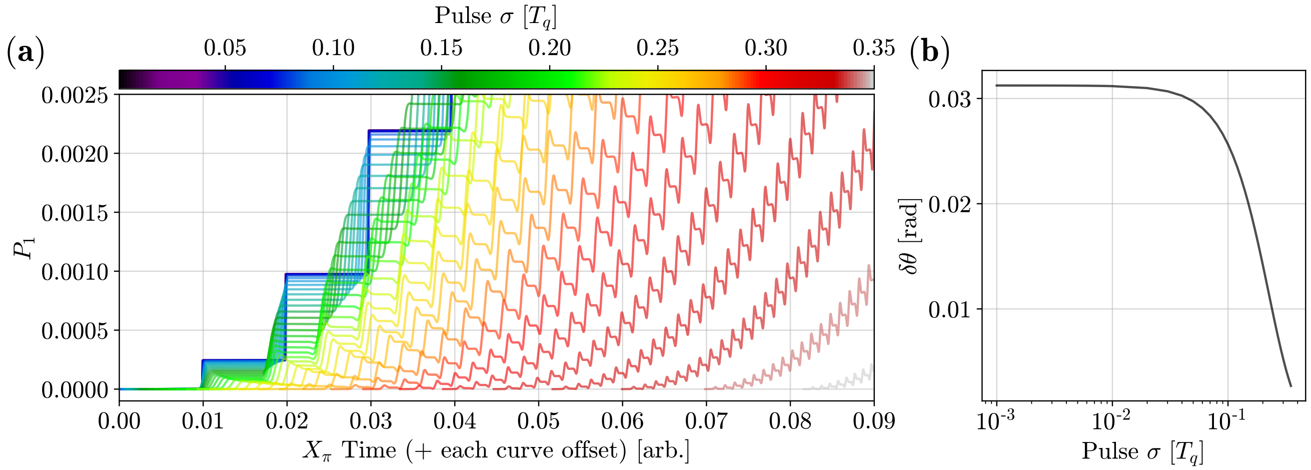

FIG. S6. (Color) **(a)** Depiction of the simulated qubit excited state evolution during delivery of the first few Gaussian pulses and as a function of the pulse σ. The x-axis is scaled to the X_π time and each curve is offset relative to their departure from the ideal delta function case of $\nu_\pi = 100$ to better show dynamics. For wide pulses ($4\sigma \sim 2\pi/\omega_{10}$) the effects of distributed delivery of the pulse energy during qubit precession can be seen as dips in P_1 at the beginning and end of the pulses. **(b)** Tip angle per pulse $\delta\theta$ (evaluated at the center of the qubit idle period) as a function of σ. Reduction in $\delta\theta$ is due primarily to pulse energy delivery when the qubit is out of phase of the target control axis, but is also affected by the decreased harmonic tone power for increasing σ. Fast pulses do not suffer from these effects and are seen to approach the Dirac delta function limit for $\sigma < 0.02T_q$.

and then anti-aligned, etc. In the anti-alignment phase the leading and trailing pulse tails induce small negative (back towards $|0\rangle$) rotations on either side of the main positive rotation; corresponding to the pulse maximum and complete alignment of $|\psi\rangle$ and the target control axis. This behavior manifests as a dip in P_1 when the pulse begins to arrive, a larger increase in P_1 as the pulse peak arrives, and another small reduction in P_1 for the trailing pulse tail.

To demonstrate these effects, Fig. S6 shows a magnified view of the qubit excited state evolution for the first few pulses and as the pulse σ is swept, as well as the reduction in $\delta\theta$ as σ increases. We find that for wide pulses ($\sigma \gtrsim 0.1$) the $X_\pi(\sigma)$ infidelity, after subtraction of the coherence limit contribution, roughly corresponds to the rotation lost due to pulse energy delivery while the qubit is anti-aligned with the desired control axis. In the main text we refer to this as the digital-pulse-only infidelity, $1-\mathcal{F}_{pulse}$. As stated in Fig 4(c), for $\sigma_{\rm JPG} = 0.19T_q$, we expect $1-\mathcal{F}_{pulse} = 1\times10^{-4}$. This is over an order of magnitude smaller than the coherence-limit contribution as we find $1-\mathcal{F}_{tot} = 2\times10^{-3}$ with finite coherence.

Leakage to the higher transmon level arises from the finite length pulse train used to evolve the qubit state. To estimate the infidelity due to higher state leakage we follow analysis in [19], which uses $\nu_\pi = 100$ and true Dirac delta function pulses. Since $(1-\mathcal{F}) \propto \nu_\pi^{-2}$ we rescale by a factor $(352/100)^{-2}$. For our qubit, with an anharmonicity $\alpha = 5\%$, this results in higher state leakage infidelity of 7×10^{-4}. We note that for our case of moderate-width pulses, the ringing in the pulse train spectrum will be further suppressed relative to the Dirac delta function pulse train in [19].

For our configuration we consider the case of pulse timing jitter when the JPG is driven with a stable external clock [19]. Gaussian-distributed thermal jitter in pulse generation in SFQ circuitry has been measured to have a standard deviation of 3 ps or less at 4.2 K [58]. However, for an $N_{JJ} = 4650$ array, the effect of thermal jitter at each JJ results only in a slight broadening of the output JPG pulse with little effect on the pulse arrival time. Timing jitter from the JPG drive signal, however, will cause jitter of the JPG output signal. Measurements of jitter in the JPG drive signal at 300 K indicate jitter of 3 ps (standard deviation), corresponding to a jitter-sourced infidelity estimate of 3×10^{-3}.

Combining all contributions above we obtain a total expected gate infidelity of $1-\mathcal{F}_{tot} = 6\times10^{-3}$, a factor of three lower than the observed JPG RB error per gate of 2×10^{-2}. We attribute the unaccounted infidelity to possible systematic and coherent errors in the RB routine and are actively investigating these effects.

VI. JPG FABRICATION DETAILS AND REDUCED I_c TEMPERATURE SENSITIVITY

Gating pulses from a JJ array with our scheme of driving with an integer number of pure sine periods means the rf dissipation is toggled rapidly between null and 100%. Depending on the I_c temperature sensitivity, this can cause an

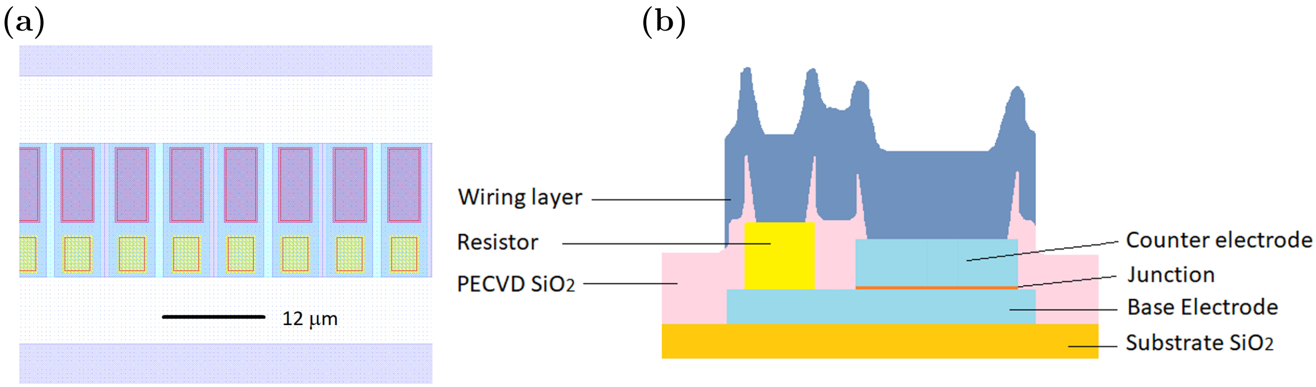

FIG. S7. (Color) **(a)** Rendering of the JJ (red) and shunt resistor R_n (yellow) layout within the center conductor of the coplanar waveguide. The top wiring layer is not shown. **(b)** Cross-sectional diagram of the JPG layer stack (non-planarized) where the view into the page for (b) corresponds to looking left along the array in (a).

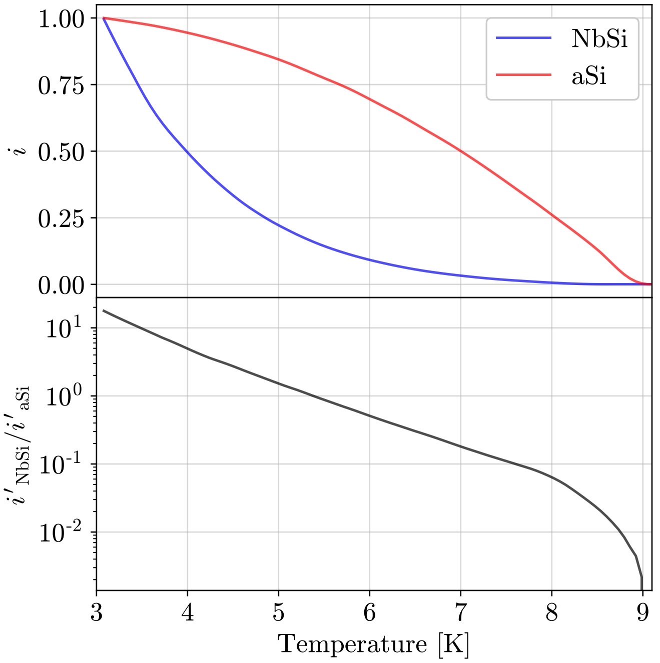

FIG. S8. (Color) Reduced critical current $i = I/I_c^{3.1\ \mathrm{K}}$ of NbSi and αSi barrier JPG devices (top), and the temperature-sensitivity ($i' = di/dT$) ratio of the NbSi and αSi devices (bottom). The drastically-reduced 3 K - 4 K temperature sensitivity of the αSi JPG provides the necessary stability of the Shapiro step when the JPG is driven with both short patterns for T_1, T_2^*, and P_{th} measurements, and with long patterns for measuring many Rabi oscillations and performing randomized benchmarking.

unstable locking range for a JJ array and make calibrations such as those detailed in Fig. 3 difficult or impossible.

To reduce the temperature sensitivity of the JPG we have departed from the superconductor-normal metal-superconductor (SNS) JJ technology [18] based on triple-stacked self-shunted niobium-doped-silicon barriers (NbSi) embedded in the center conductor of a niobium (Nb) superconducting coplanar waveguide. Instead, we form the JPG array from externally shunted superconductor-insulator-superconductor (SIS) junctions, as can be seen in Fig. S7(a). The JJs were fabricated using amorphous silicon (αSi) for the barriers and palladium-gold (PdAu) alloy for shunting resistors. Due to the small value of the shunting resistors (25 mΩ) required to achieve the target characteristic frequency of $f_c = 10$ GHz, the usual method of fabricating planar resistors could not be used. That method relies on resistor films with sheet resistances of 1–2 $\Omega/\Box$. The form factor to achieve 25 mΩ entails very wide and long arrays to accommodate interconnecting vias to minimize contact resistance. Instead, (vertical) stud resistors were used with a height similar to the junction counter electrode thickness. See a schematic of the cross-section in Fig. S7(b). This

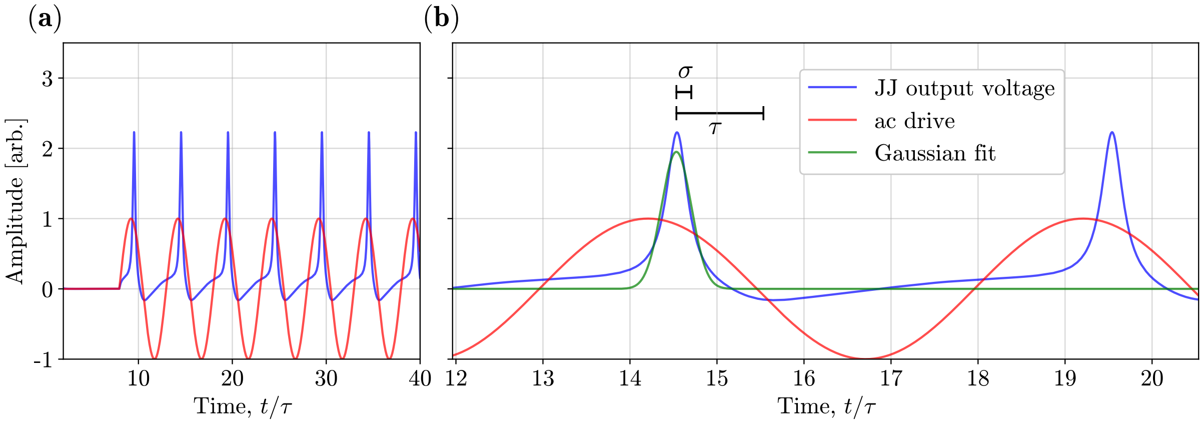

FIG. S9. (Color) Simulation of the output of a single-JJ using the RSJ model, driven by an ac drive at frequency $f_d/f_c = 0.2$ and with a dc current bias which places the JJ on the first Shapiro step and results in a periodic train of SFQ pulses. **(a)** The first seven SFQ pulses and the ac drive signal **(b)** Magnification of the second and third SFQ pulses with a Gaussian fit to the former. Here we also show pictorially the relative size of the fitted Gaussian σ and the JJ characteristic time τ.

enables significant reduction in the JJ cell size and results in a much more compact array.

The circuits were fabricated on 3-inch silicon wafers with 150 nm of thermal oxide. A trilayer of Nb/αSi/Nb was deposited by sputtering. The thickness of the silicon was chosen to obtain a junction critical current density of 12 kA/cm^2. PdAu resistors were electron-beam evaporated with a thickness of 240 nm. Silicon oxide insulator was then deposited by electron cyclotron plasma enhanced chemical vapor deposition (EC-PECVD). Lithography followed by dry etching opened vias to the junction counter electrodes, resistors, and base electrodes. The wiring layer was deposited by niobium sputtering and then patterned by dry etching. Resulting devices display $I_c = 3.05$ mA and $R_n = 6.93$ mΩ, giving $f_c = 10.2$ GHz. These devices exploit the much more stable I_c of αSi barrier JJs with respect to temperature. Fig S8 shows a comparison of the temperature stability of I_c for NbSi and αSi devices, demonstrating that in our region of interest of 3–3.3 K the αSi barrier device is 10–20 times less sensitive.

VII. RELATION BETWEEN JPG τ AND GAUSSIAN-FITTED σ

Simulations of JPG dynamics can be performed by numerically integrating the resistively shunted junction (RSJ) model with an ac drive term $i_{\text{ac}} \sin(f_d \Theta / f_c)$

$$\frac{d\phi}{d\Theta^2} = \frac{1}{\beta_c}\left[i + i_{\text{ac}} \sin\left(\frac{f_d}{f_c}\Theta\right) - \sin\phi - \frac{d\phi}{d\Theta}\right]. \tag{S3}$$

Here ϕ is the superconducting phase difference across the JJ, Θ is the dimensionless time variable $\Theta = (2\pi I_c R_n/\Phi_0)t = 2\pi t/\tau$, $\beta_c = 2\pi f_c R_n C$ is the Stewart-McCumber damping parameter with C the intrinsic JJ shunting capacitance, and $i = I_d/I_c$ and $i_{\text{ac}} = I_{\text{ac}}/I_c$ are the normalized dc and ac (amplitude) bias currents, respectively. Note that the ac drive frequency f_d has units of time scaled to the Josephson time constant $\tau = 1/f_c = \Phi_0/I_c R_n$ and not to Θ, as these differ by a factor of 2π.

With the second Josephson equation

$$V(t) = \frac{\Phi_0}{2\pi}\frac{d\phi}{dt} \tag{S4}$$

and Eq. S3, we can solve for the time-dependent voltage response of the JJ. We choose $i_{\text{ac}} = 0.6$, and $f_d/f_c = 0.2$, which approximately reflects the relation between the JPG f_c of 10.2 GHz and the typical drive frequency of $\omega_d/2\pi = 2.68$ GHz, and ramp i_{dc} until pulses are observed. For $\beta_c \ll 1$, the JJ response is overdamped and non-hysteretic so we simulate a heavily overdamped regime of $\beta_c = 0.01$. For our JPG, $\beta_c \lesssim 10^{-4}$. Results are displayed in Fig S9 and we perform a Gaussian fit to one SFQ pulse. Time units are re-scaled from the Θ time of Eq S3 to units of the JJ characteristic time τ. For these parameters we extract $\sigma/\tau = 1.08/2\pi$. This indicates that, given the JPG τ of 98 ps, the pulses at 3 K (on-chip, prior to any significant dispersion and broadening) have an expected Gaussian sigma of 17 ps. Fits at room temperature of the JPG pulses captured on the oscilloscope (Sec. III B of main body) thus display

approximately a factor of two in broadening compared to the expected on-chip value. As mentioned in Sec. III B, this is the reason we take $\sigma = 35$ ps to be a conservative upper bound on the approximate width of pulses delivered to the qubit, even after traversal of the readout cavity resonance.
